# Nomograms to predict tumor regression grade (TRG) and ypTNM staging in patients with locally advanced esophageal cancer receiving neoadjuvant therapy

**DOI:** 10.1186/s12957-024-03474-7

**Published:** 2024-07-27

**Authors:** Jianhao Qiu, Zhan Zhang, Junjie Liu, Yue Zhao, Yongmeng Li, Zhanpeng Tang, Lin Li, Yu Tian, Hui Tian

**Affiliations:** 1 Department of Thoracic Surgery, Qilu Hospital of Shandong University, Jinan, Shandong China; 2 Department of Thoracic Surgery, Qianfoshan Hospital in the Shandong Province, Jinan, Shandong China

**Keywords:** Esophageal cancer, Neoadjuvant therapy, Immunotherapy, Neoadjuvant pathologic TNM, Tumor regression grade, Nomogram

## Abstract

**Background:**

Neoadjuvant therapy (NT) has increased survival rates for patients with locally advanced esophageal cancer (EC), but estimating the impact of NT treatment prior to surgery is still very difficult.

**Methods:**

A retrospective study of the clinical information of 150 patients with locally advanced EC who got NT at Qilu Hospital of Shandong University between June 2018 and June 2023. Patients were randomized into training and internal validation groups at a 3:1 ratio. Furthermore, an external validation cohort comprised 38 patients who underwent neoadjuvant therapy at Qianfoshan Hospital in the Shandong Province between June 2021 and June 2023. Independent risk factors were identified using univariate and multivariate logistic regression (forward stepwise regression). Predictive models and dynamic web nomograms were developed by integrating these risk factors.

**Results:**

A total of 188 patients with locally advanced EC were enrolled, of whom 118 achieved stage I of neoadjuvant pathologic TNM (ypTNM) after receiving NT and 129 achieved grades 0-1 in the tumor regression grade (TRG). Logistic regression analysis identified five independent predictors of TRG grades 0-1: pulmonary function tests (PFT), prognostic nutritional index (PNI), triglyceride (TG) levels, squamous cell carcinoma antigen (SCC-Ag) levels, and combination immunotherapy. The areas under the receiver operating characteristic (ROC) curves for the training, internal validation, and external validation groups were 0.87, 0.75, and 0.80, respectively. Meanwhile, two independent predictors of stage I of ypTNM were identified: prealbumin (PA) and SCC antigen. The areas under the ROC curves for the training, internal validation, and external validation groups were 0.78, 0.67, and 0.70, respectively. The Hosmer-Lemeshow test for both predictive models showed excellent calibration, with well-fitted calibration curves. Decision curve analysis (DCA) and clinical impact curves (CIC) have demonstrated that nomograms are of clinical utility.

**Conclusion:**

The nomograms performed well in predicting the likelihood of stage I of ypTNM and TRG grade 0-1 after NT in patients with locally advanced EC. It helps thoracic surgeons to predict the sensitivity of patients to NT before surgery, which enables precise treatment of patients with locally advanced EC.

**Supplementary Information:**

The online version contains supplementary material available at 10.1186/s12957-024-03474-7.

## Introduction

Esophageal cancer (EC), a prevalent malignant tumor, ranks seventh in incidence and sixth in mortality among all cancers. In 2020, it accounted for one in every 18 cancer deaths [1].The prognosis of this disease is poor, characterized by a five-year survival rate below 20%, largely due to the fact that the majority of patients have locally advanced disease when first diagnosed [2]. EC primarily presents as two subtypes: squamous cell carcinoma and adenocarcinoma. Squamous cell carcinoma comprises 90% of cases worldwide, though adenocarcinoma incidence has recently surpassed squamous cell carcinoma in developed countries [3]. Surgery is the preferred treatment for resectable cases [4]. Unfortunately, over half of the patients are diagnosed at advanced stages, limiting treatment options [5]. For locally advanced EC, the standard treatment combines neoadjuvant therapy (NT) with esophagectomy [6]. Preoperative NT has been shown to improve long-term survival and reduce local recurrence [7, 8]. Recent phase III clinical trials indicate that adding immunotherapy to preoperative neoadjuvant chemoradiotherapy extends disease-free survival compared to radiotherapy alone [9]. Only about 30% of patients achieve pathologic complete remission (pCR) with NT, and these patients benefit more from NT, but those patients who are not sensitive to NT may not undergo surgery due to disease progression during treatment [10–12]. However, the main problem at present is that we cannot accurately predict the efficacy of NT, and we cannot timely screen these patients who can achieve pCR or who can realize the downgrading of pathological staging. An accurate method to predict the efficacy of NT would be crucial in developing personalized treatment plans.

The main pathological standards for assessing the efficacy of neoadjuvant therapy for EC are neoadjuvant pathologic TNM (ypTNM) stage and tumor regression grade (TRG). The 8th edition of the American Joint Committee on Cancer (AJCC) Cancer Staging Manual endorses ypTNM staging for post-neoadjuvant pathological evaluation in EC patients [13]. Research indicates that ypTNM staging more accurately predicts patient prognosis post-neoadjuvant therapy than traditional pathologic TNM (pTNM) staging [14, 15]. One study showed that the survival of patients with ypTNM stages III-IV did not differ from that of patients with pTNM stages III-IV. Patients with ypTNM stages I-II had a shorter but better survival than patients with pTNM stages I-II than patients with pTNM stage III [16]. This may be due to the downstaging effect of neoadjuvant therapy, which suggests that for patients with locally advanced disease the ability to achieve a downstaging effect of ypTNM stage I-II after neoadjuvant therapy suggests that patients have a better prognosis [17].

Another key evaluation criterion is TRG, which the College of American Pathologists classifies into four grades for tumor regression (the percentage of cancer cells remaining at the primary site) after preoperative NT for EC: grade 0 indicates complete response with no surviving cancer cells; grade 1, a moderate response with single or small clusters of residual cancer cells; grade 2, a mild response characterized by residual cancer foci amidst extensive interstitial fibrosis; and grade 3, no response, indicated by minimal or no cancer cell necrosis and a high volume of remaining cancer cells [18]. Several studies have demonstrated that TRG is a more precise prognostic predictor than ypTNM after NT [19, 20]. Clinical trials have also shown significantly improved survival rates in patients with no residual tumor cells at the primary site postoperatively [10, 21, 22].

In this study, we explored potential indicators linked to the efficacy of NT in EC patients. Additionally, we developed nomograms to predict ypTNM and TRG grades post-NT in patients with locally advanced EC, based on clinical characteristics and laboratory tests. The clinical effectiveness of these nomograms were subsequently evaluated.

### Patients and methods

#### Patients’ selection

This retrospective study included 150 patients with EC who received preoperative NT between June 2018 and June 2023 at Qilu Hospital of Shandong University. The study inclusion criteria were as follows: 1) according to the 8th edition of the Clinical Staging Guidelines for Esophageal Cancer, patients were evaluated as clinical stage III EC by enhanced computed tomography (CT), endoscopic ultrasound, or positron emission tomography–computed tomography (PET-CT); 2) received complete preoperative NT; and 3) open or thoracoscopic tumor resection at least 3 weeks after completion of NT; 4) having complete clinicopathological data. In addition, 38 patients who met the inclusion criteria were included as an independent external validation cohort by searching the thoracic surgery database of Qianfoshan Hospital in the Shandong Province according to the inclusion criteria. The study was approved by the Medical Ethics Committee of our institute, and all patients signed a written informed consent.

#### Data collection

We collected the following patient data as required for the study: 1) baseline data such as height, weight, and gender were collected; 2) patients' preoperative pulmonary function test results; 3) routine blood and laboratory test results such as liver function, kidney function, nutritional status, lipid levels, and tumor markers before surgery; 4) NT strategies; and 5) patients' tumor location, and histological type.

#### Patients follow-up

All patients were followed up in our outpatient department every three months for the first two years post-surgery, and subsequently every six months. During each scheduled outpatient visit, routine neck, thoracic and abdominal computed tomography (CT) scans were performed for surveillance. In cases of neurological symptoms, cranial CT scans or magnetic resonance imaging (MRI) were conducted. If feasible, positron emission tomography (PET)-CT scans were recommended. Overall Survival (OS) is defined as the interval from the date of surgery to the date of death or last follow-up. Disease-Free Survival (DFS) is defined as the time from the date of surgery to the date of disease recurrence or death from any cause. The primary endpoint was the 1-year and 3-year rates of DFS, and the secondary endpoints were the 1-year and 3-year rates of OS.

#### Treatment

All patients received neoadjuvant therapy. All NT protocols are formulated using the esophageal cancer treatment guidelines of the Chinese Society of Clinical Oncology and the National Comprehensive Cancer Network as the standard. The treatment used in patients receiving neoadjuvant chemotherapy was a 3-cycle regimen of cisplatin (75 mg/m^2^, d1, q3w) in combination with paclitaxel (175 mg/m^2^, d1, q3w). Patients enrolled in immunotherapy were treated with pembrolizumab (200 mg) or karelizumab (200 mg) every 3 weeks and maintained for 3 cycles. Three weeks following the conclusion of NT, all patients had neck, thoracic, and abdomen contrast-enhanced CT scans as well as ultrasonography endoscopy. Patients were hospitalized for surgery (Ivor Lewis, McKeown, or Sweet esophagectomies) if our thoracic surgery team determined they were candidates for radical esophagectomy. Each patient underwent thorough clearing of mediastinal lymph nodes, including bilateral recurrent laryngeal nerve lymph nodes, in order to precisely examine the state of the lymph nodes. Patients whose neck lymph nodes were suspected of being positive had a three-field lymph node dissection.

#### Histological assessment

All pathological specimens underwent fixation in formalin, followed by sectioning and staining with hematoxylin-eosin, adhering to standard pathology section preparation protocols. Two experienced pathologists, blinded to patient data, independently assessed each tissue section using light microscopy for histopathological evaluation. The outcome of patients after NT was evaluated using ypTNM as well as TRG. The eighth edition of the TNM staging of esophageal cancer, published by the AJCC and the Union for International Cancer Control (UICC), was used to appraise the ypTNM stage, whereas the College of American Pathologists' criteria were used to evaluate TRG^13, 18^.

#### Nomogram construction

Univariate logistic regression analyses were conducted to identify factors influencing TRG and ypTNM stage of NT in EC patients. Factors demonstrating a P-value less than 0.20 in these analyses were subsequently included in multivariate analyses. Predictive models were constructed based on independent risk factors identified through multivariate logistic regression analysis, with a significance threshold of P < 0.05. The results of the multivariable logistic regression models were then used to construct nomogram. These models were utilized to compute scores for each variable, enabling the prediction of TRG and ypTNM stage through the aggregation of these scores.

#### Nomogram performance

The predictive nomogram’s performance was assessed based on discrimination, calibration, and clinical utility. Receiver operating characteristic (ROC) curves were used to assess the discriminatory effectiveness of the predicted nomogram [23].The calibration measures how well the predicted probabilities match the true results. The Hosmer-Lemeshow test was used to assess the calibration ability, and a P-value >0.05 indicated satisfactory calibration [24]. Calibration was then assessed further by constructing a nomogram calibration plot. Internal and external verifications were conducted using the bootstrapping method with 1000 repetitions [25]. Decision curve analysis (DCA) and clinical impact curves (CIC) were conducted to evaluate the clinical utility of the predictive nomograms, focusing on the net benefit across various probability thresholds [26, 27].

#### Statistical analysis

Kaplan–Meier analyses were performed to compare patients’ survival outcomes. Continuous variables that were normally distributed were analyzed using the t-test and expressed as the mean ± standard deviation (SD). For non-normally distributed continuous variables, data were presented as medians with interquartile ranges (IQR) and compared between groups using the Mann-Whitney U test. Categorical variables were assessed using Pearson's chi-squared test or Fisher's exact test, as appropriate. Statistical significance was established at a two-sided P-value of less than 0.05. Data analyses were conducted using SPSS software (version 25.0, IBM Corp., Armonk, NY, USA) and the R Project software (version 4.2.1, http://www.R-project.org).

## Results

### Patient characteristics

**Fig. 1:**
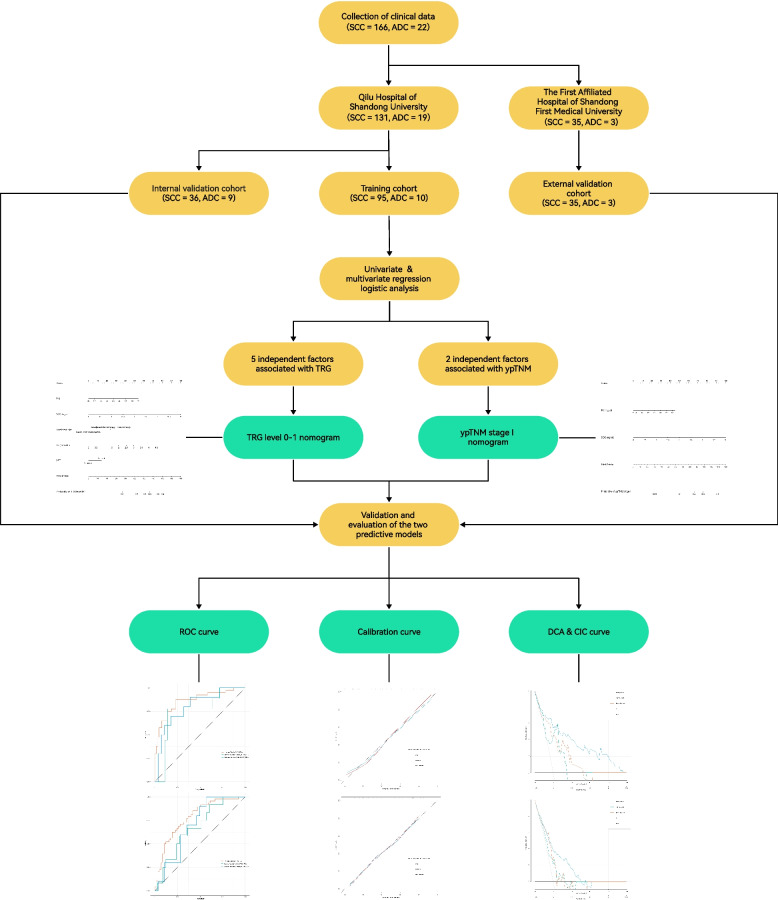
Flow diagram of patient selection through the study. SCC: squamous cell carcinoma; ADC: adenocarcinoma; TRG: tumor regression grade; ypTNM: neoadjuvant pathologic TNM; ROC: receiver operating characteristic; DCA: decision curve analysis; CIC: clinical impact curves

The detailed process is shown in Fig. 1. The study included a total of 188 patients with EC, with 150 patients from Qilu Hospital of Shandong University and 38 patients from Qianfoshan Hospital in the Shandong Province. All participants satisfied the inclusion and exclusion criteria. A total of 59 patients (31.4%) achieved a TRG grade of 0-1, while 70 patients (37.2%) were classified as Stage I (Table 1). A total of 150 patients from Qilu Hospital of Shandong University were randomly divided into two groups, namely the training cohort and the internal validation cohort, in a ratio of 7:3; patients from Qianfoshan Hospital in the Shandong Province formed the external validation cohort. As shown in Table 1, there were no statistically significant differences between the validation and training cohorts with regard to variables. The characteristics of both cohorts are detailed in Tables 2 and 3.

**Table 1:** Preoperative baseline characteristics of included patients and comparison between groups

**Characteristics**	**TC**	**IVC**	**EVC**	*P* value^*^	
	**(*n*=105)**	**(*n*=45)**	**(*n*=38)**	**TC vs IVC **	**TC vs EVC**
**Demographics**					
Age (years), M (Q₁, Q₃)	63.00 (58.00 - 67.00)	64.00 (58.00 - 67.00)	64.00 (58.00 - 70.00)	0.60	0.43
Gender, n(%)				0.50	0.25
Female	16 (15.24)	5 (11.11)	3 (7.89)		
Male	89 (84.76)	40 (88.89)	35 (92.11)		
BMI (kg/m^2^), M (Q₁, Q₃)	22.34 (20.90 - 23.67)	26.96 (26.33 - 28.31)	22.72 (20.78 - 24.48)	<0.01	0.43
Smoking history, n(%)				0.71	0.62
No	34 (32.38)	16 (35.56)	14 (36.84)		
Yes	71 (67.62)	29 (64.44)	24 (63.16)		
Alcohol use, n (%)				0.42	0.50
No	35 (33.33)	12 (26.67)	15 (39.47)		
Yes	70 (66.67)	33 (73.33)	23 (60.53)		
Family history of tumors, n (%)				0.74	<0.01
No	101 (96.19)	42 (93.33)	30 (78.95)		
Yes	4 (3.81)	3 (6.67)	8 (21.05)		
PFT, N(%)				0.94	0.92
Normal	59 (56.19)	25 (55.56)	21 (55.26)		
Abnormal	46 (43.81)	20 (44.44)	17 (44.74)		
**Complete blood count**					
Hb (g/L), M (Q₁, Q₃)	129.00 (118.00 - 137.00)	132.00 (120.00 - 140.00)	129.50 (120.50 - 135.50)	0.42	0.83
dNLR, M (Q₁, Q₃)	1.39 (1.08 - 1.90)	1.27 (1.10 - 1.89)	1.70 (1.28 - 2.36)	0.77	0.04
NLR, M (Q₁, Q₃)	2.02 (1.41 - 2.62)	1.86 (1.47 - 2.50)	2.49 (1.83 - 3.33)	0.99	0.02
PLR, M (Q₁, Q₃)	132.69 (106.86 - 173.94)	142.27 (102.63 - 182.86)	154.13 (117.50 - 173.32)	0.74	0.21
LMR, M (Q₁, Q₃)	3.26 (2.49 - 3.98)	3.24 (2.12 - 4.21)	2.63 (2.26 - 3.39)	0.78	0.04
SII, M (Q₁, Q₃)	436.43 (288.46 - 590.82)	404.00 (255.15 - 581.88)	525.99 (339.32 - 792.99)	0.85	0.04
**Liver & renal function**					
TBil (μmol/L), M (Q₁, Q₃)	8.70 (7.30 - 11.10)	9.40 (7.60 - 12.70)	10.30 (8.65 - 12.83)	0.29	0.02
ALT (U/L), M (Q₁, Q₃)	16.00 (13.00 - 25.00)	14.00 (10.00 - 20.00)	16.00 (11.25 - 26.00)	0.04	0.96
AST (U/L), M (Q₁, Q₃)	19.00 (16.00 - 23.00)	17.00 (13.00 - 22.00)	18.50 (14.25 - 23.75)	0.11	0.60
LDH (U/L), M (Q₁, Q₃)	196.00 (174.00 - 213.00)	192.00 (168.00 - 214.00)	214.50 (181.25 - 235.50)	0.59	0.01
Urea (mmol/L), M (Q₁, Q₃)	5.60 (4.50 - 6.50)	5.10 (4.20 - 6.40)	5.20 (4.60 - 5.85)	0.22	0.29
Cr (μmol/L), M (Q₁, Q₃)	71.00 (62.00 - 80.00)	70.00 (65.00 - 80.00)	68.50 (60.00 - 76.00)	0.94	0.17
UA (μmol/L), M (Q₁, Q₃)	299.00 (255.00 - 359.00)	305.00 (275.00 - 367.00)	302.50 (251.00 - 347.25)	0.34	0.61
**Nutritional status**					
Glu (mmol/L), M (Q₁, Q₃)	5.13 (4.71 - 5.45)	5.23 (4.94 - 5.93)	5.12 (4.62 - 5.90)	0.09	0.54
TP (g/L), M (Q₁, Q₃)	67.80 (64.40 - 71.30)	67.40 (63.70 - 71.70)	66.30 (63.15 - 71.25)	0.77	0.20
ALB (g/L), M (Q₁, Q₃)	43.00 (40.50 - 45.40)	41.70 (40.70 - 44.00)	41.15 (39.75 - 44.33)	0.30	0.12
PA (g/L), M (Q₁, Q₃)	24.00 (21.30 - 26.50)	23.50 (20.60 - 27.90)	21.50 (19.55 - 24.15)	0.78	0.02
PNI, M (Q₁, Q₃)	50.95 (47.80 - 53.70)	49.25 (46.35 - 52.80)	48.50 (45.86 - 53.05)	0.22	0.06
**Lipid levels**					
Cho (mmol/L), M (Q₁, Q₃)	4.96 (4.34 - 5.61)	4.74 (4.23 - 5.45)	4.25 (4.00 - 4.86)	0.68	<0.01
HDL (mmol/L), M (Q₁, Q₃)	1.25 (1.04 - 1.48)	1.11 (1.02 - 1.26)	1.14 (0.99 - 1.39)	0.01	0.13
LDL (mmol/L), M (Q₁, Q₃)	2.97 (2.48 - 3.51)	2.96 (2.51 - 3.55)	2.43 (2.07 - 2.81)	0.95	<0.01
TG (mmol/L), M (Q₁, Q₃)	1.14 (0.91 - 1.52)	1.48 (1.01 - 2.43)	1.12 (0.91 - 1.95)	0.01	0.58
**Tumor Biomarkers**					
CEA (ng/ml), M (Q₁, Q₃)	2.44 (1.58 - 3.35)	2.16 (1.42 - 2.79)	3.23 (2.42 - 5.53)	0.19	<0.01
SCC-Ag (ng/ml), M (Q₁, Q₃)	1.20 (0.88 - 1.93)	1.20 (0.81 - 1.70)	0.85 (0.60 - 1.55)	0.24	0.04
CA-199 (U/ml), M (Q₁, Q₃)	9.12 (6.12 - 13.80)	9.68 (5.92 - 17.70)	6.04 (4.18 - 8.40)	0.66	<0.01
CA-125 (U/ml), M (Q₁, Q₃)	8.50 (5.81 - 11.40)	9.12 (5.75 - 13.20)	9.19 (6.50 - 11.78)	0.62	0.38
CA-724 (U/ml), M (Q₁, Q₃)	3.39 (1.85 - 6.19)	3.20 (2.32 - 5.15)	3.01 (1.64 - 5.71)	0.86	0.30
**Tumor-related characteristics**					
Location, n (%)				0.05	0.80
Upper thoracic segment	4 (3.81)	0 (0.00)	2 (5.26)		
Middle thoracic segment	48 (45.71)	13 (28.89)	16 (42.11)		
Lower thoracic segment	53 (50.48)	32 (71.11)	20 (52.63)		
Histology, n (%)				0.08	0.99
Adenocarcinoma	10 (9.52)	9 (20.00)	3 (7.89)		
Squamous carcinoma	95 (90.48)	36 (80.00)	35 (92.11)		
**Treatment plan, n (%)**				0.11	0.02
NC	65 (61.90)	34 (75.56)	15 (39.47)		
NC+ immunotherapy	40 (38.10)	11 (24.44)	23 (60.53)		
**Outcome**					
TRG, n (%)				0.03	0.19
Level 0-1	40 (38.10)	9 (20.00)	10 (26.32)		
Level 2-3	65 (61.90)	36 (80.00)	28 (73.68)		
ypTNM, n (%)				0.05	0.04
Stage I	48 (45.71)	12 (26.67)	10 (26.32)		
Stage II-IVA	57 (54.29)	33 (73.33)	28 (73.68)		

*TC* Training cohort, *IVC* Internal validation cohort, *EVC* External validation cohort, *BMI* Body mass index, *PFT* Pulmonary function tests, *dNLR* derived neutrophils/ (leukocytes minus neutrophils) ratio, *NLR* Neutrophil–lymphocyte ratio, *PLR* Platelet–lymphocyte ratio, *LMR* Lymphocyte-to-monocyte ratio, *SII* Systemic immune-inflammation index, *TBil* Total bilirubin, *ALT* Alanine aminotransferase, *AST* Aspartate aminotransferase, *LDH* Lactate dehydrogenase, *Cr* Creatinine, *UA* Uric acid, *Glu* Glucose, *TP* Total protein, *ALB* Albumin, *PA* Prealbumin, *PNI* Prognostic nutritional index, *Cho* Cholesterol, *HDL* High-density lipoprotein, *LDL* Low-density lipoprotein, *TG* Triglyceride, *CEA* Carcinoembryonic antigen, *SCC-Ag* Squamous cell carcinoma antigen, *CA-199* Carbohydrate antigen 199, *CA-125* Carbohydrate antigen 125, *CA-724* Carbohydrate antigen 724, *NC* Neoadjuvant chemotherapy, *TRG* Tumor regression grade

^*^P-value for the comparison between training cohort and validation cohort (internal validation cohort and external validation cohort)

**Table 2:** Preoperative clinical characteristics of patients with TRG level 0–1 and 2–3 in the training and validation cohorts (internal and external)

Characteristics	Training Cohort			Internal Validation Cohort			External Validation Cohort		
	TRG levels 0–1 (*n*=40)	TRG levels 2-3 (*n*=65)	*P* value	TRG levels 0–1 (*n*=9)	TRG levels 2-3 (*n*=36)	*P* value	TRG levels 0–1 (*n*=10)	TRG levels 2-3 (*n*=28)	*P* value
**Demographics**									
Age (years), M (Q₁, Q₃)	61.50 (57.00 - 66.00)	63.00 (59.00 - 68.00)	0.12	62.00 (60.00 - 70.00)	64.00 (58.00 - 66.25)	0.91	64.00 (60.00 - 69.50)	63.00 (57.75 - 70.25)	0.56
Gender, n(%)			0.96			0.99			0.55
Female	6 (15.00)	10 (15.38)		1 (11.11)	4 (11.11)		0 (0.00)	3 (10.71)	
Male	34 (85.00)	55 (84.62)		8 (88.89)	32 (88.89)		10 (100.00)	25 (89.29)	
BMI (kg/m2), M (Q₁, Q₃)	22.14 (21.07 - 23.48)	22.47 (20.83 - 23.68)	0.78	26.96 (26.53 - 28.06)	27.02 (26.25 - 28.32)	0.97	20.97 (19.58 - 22.75)	23.25 (21.20 - 24.92)	0.10
Smoking history, n(%)			0.65			0.99			0.06
No	14 (35.00)	20 (30.77)		3 (33.33)	13 (36.11)		1 (10.00)	13 (46.43)	
Yes	26 (65.00)	45 (69.23)		6 (66.67)	23 (63.89)		9 (90.00)	15 (53.57)	
Alcohol use, n (%)			0.76			0.45			<0.01
No	14 (35.00)	21 (32.31)		1 (11.11)	11 (30.56)		0 (0.00)	15 (53.57)	
Yes	26 (65.00)	44 (67.69)		8 (88.89)	25 (69.44)		10 (100.00)	13 (46.43)	
Family history of tumors, n (%)			0.31			0.50			0.41
No	37 (92.50)	64 (98.46)		8 (88.89)	34 (94.44)		7 (70.00)	23 (82.14)	
Yes	3 (7.50)	1 (1.54)		1 (11.11)	2 (5.56)		3 (30.00)	5 (17.86)	
PFT, N(%)			0.07			0.71			0.99
Normal	27 (67.50)	32 (49.23)		6 (66.67)	19 (52.78)		6 (60.00)	15 (53.57)	
Abnormal	13 (32.50)	33 (50.77)		3 (33.33)	17 (47.22)		4 (40.00)	13 (46.43)	
**Complete blood coun**t									
Hb (g/L), M (Q₁, Q₃)	127.00 (115.25 - 135.00)	130.00 (121.00 - 140.00)	0.11	131.00 (120.00 - 140.00)	132.50 (119.75 - 140.25)	0.68	128.00 (119.25 - 132.25)	131.00 (121.50 - 137.00)	0.34
dNLR, M (Q₁, Q₃)	1.54 (1.25 - 1.92)	1.29 (1.03 - 1.82)	0.09	1.76 (1.32 - 2.62)	1.23 (0.98 - 1.71)	0.04	2.18 (1.75 - 2.61)	1.51 (1.06 - 2.15)	0.06
NLR, M (Q₁, Q₃)	2.09 (1.77 - 2.89)	1.85 (1.38 - 2.49)	0.13	2.41 (2.11 - 5.65)	1.73 (1.36 - 2.40)	0.02	3.22 (3.00 - 3.76)	2.07 (1.75 - 3.24)	0.07
PLR, M (Q₁, Q₃)	132.63 (104.15 - 174.77)	132.69 (108.92 - 173.65)	0.86	155.38 (102.63 - 200.00)	140.54 (102.02 - 182.62)	0.67	156.27 (107.97 - 182.68)	151.98 (120.04 - 170.78)	0.96
LMR, M (Q₁, Q₃)	3.28 (2.44 - 3.92)	3.26 (2.56 - 4.16)	0.66	2.03 (1.19 - 3.84)	3.32 (2.55 - 4.61)	0.20	2.41 (1.93 - 2.87)	2.89 (2.40 - 3.63)	0.13
SII, M (Q₁, Q₃)	420.41 (289.80 - 593.01)	440.54 (288.46 - 590.82)	0.87	481.18 (317.62 - 675.00)	377.71 (240.41 - 574.93)	0.27	657.23 (450.72 - 746.71)	505.90 (281.08 - 817.10)	0.29
**Liver & renal function**									
TBil (μmol/L), M (Q₁, Q₃)	8.70 (5.97 - 10.55)	8.80 (7.50 - 11.50)	0.35	10.20 (7.80 - 11.90)	9.35 (7.55 - 12.77)	0.65	10.10 (7.90 - 12.32)	10.30 (8.75 - 12.90)	0.74
ALT (U/L), M (Q₁, Q₃)	18.00 (11.75 - 26.00)	16.00 (14.00 - 21.00)	0.58	14.00 (8.00 - 19.00)	15.00 (10.75 - 20.00)	0.69	25.50 (16.00 - 28.25)	15.00 (10.00 - 20.50)	0.05
AST (U/L), M (Q₁, Q₃)	20.00 (17.00 - 23.75)	18.00 (15.00 - 22.00)	0.17	16.00 (14.00 - 22.00)	18.00 (13.00 - 22.00)	0.69	24.00 (19.50 - 30.50)	16.00 (14.00 - 20.25)	<0.01
LDH (U/L), M (Q₁, Q₃)	198.50 (172.75 - 225.75)	191.00 (174.00 - 207.00)	0.26	214.00 (168.00 - 234.00)	189.50 (168.00 - 212.25)	0.34	223.50 (207.00 - 235.50)	208.50 (174.75 - 230.50)	0.35
Urea (mmol/L), M (Q₁, Q₃)	5.33 (4.18 - 6.54)	5.60 (4.70 - 6.50)	0.50	4.50 (4.20 - 6.41)	5.10 (4.35 - 6.31)	0.48	5.90 (4.62 - 7.05)	5.15 (4.42 - 5.60)	0.25
Cr (μmol/L), M (Q₁, Q₃)	70.00 (61.50 - 80.25)	71.00 (63.00 - 80.00)	0.57	68.00 (66.00 - 76.00)	71.00 (65.00 - 80.25)	0.96	70.00 (60.75 - 80.75)	68.00 (58.50 - 75.25)	0.40
UA (μmol/L), M (Q₁, Q₃)	304.50 (248.25 - 368.75)	299.00 (264.00 - 351.00)	0.91	336.00 (301.00 - 355.00)	299.50 (271.00 - 367.75)	0.39	338.50 (285.50 - 362.25)	282.50 (240.00 - 339.75)	0.16
**Nutritional status**									
Glu (mmol/L), M (Q₁, Q₃)	5.23 (4.63 - 5.61)	5.10 (4.75 - 5.43)	0.42	5.23 (4.98 - 5.77)	5.20 (4.77 - 5.96)	0.74	4.83 (4.40 - 5.47)	5.16 (4.81 - 5.96)	0.11
TP (g/L), M (Q₁, Q₃)	66.80 (64.50 - 70.92)	68.10 (64.40 - 71.50)	0.51	68.50 (65.00 - 69.90)	67.05 (63.70 - 72.50)	0.94	68.20 (66.80 - 71.50)	64.70 (62.12 - 70.75)	0.10
ALB (g/L), M (Q₁, Q₃)	42.55 (40.65 - 44.80)	43.10 (40.40 - 45.40)	0.62	42.80 (41.50 - 44.70)	41.55 (40.60 - 43.78)	0.46	42.65 (41.12 - 45.17)	40.95 (39.22 - 42.75)	0.08
PA (g/L), M (Q₁, Q₃)	24.55 (22.58 - 26.13)	23.70 (19.20 - 27.50)	0.57	27.10 (20.60 - 29.10)	23.20 (20.67 - 26.88)	0.36	20.65 (19.88 - 22.02)	21.85 (19.25 - 25.05)	0.48
PNI, M (Q₁, Q₃)	49.62 (47.71 - 52.16)	51.30 (48.10 - 54.90)		48.70 (45.95 - 51.90)	49.40 (46.80 - 52.95)	0.37	48.42 (46.66 - 53.70)	48.50 (45.55 - 50.99)	0.50
**Lipid levels**									
Cho (mmol/L), M (Q₁, Q₃)	5.17 (4.47 - 5.67)	4.84 (4.30 - 5.39)	0.29	4.32 (4.26 - 5.45)	4.77 (4.22 - 5.48)	0.97	4.08 (3.65 - 5.06)	4.30 (4.03 - 4.83)	0.48
HDL (mmol/L), M (Q₁, Q₃)	1.17 (1.02 - 1.37)	1.30 (1.07 - 1.50)	0.14	1.08 (1.04 - 1.39)	1.12 (1.02 - 1.24)	0.74	1.14 (1.07 - 1.38)	1.14 (0.96 - 1.38)	0.50
LDL (mmol/L), M (Q₁, Q₃)	3.10 (2.57 - 3.76)	2.92 (2.40 - 3.42)	0.28	3.02 (2.52 - 3.27)	2.91 (2.47 - 3.59)	0.94	2.38 (1.98 - 2.72)	2.43 (2.08 - 2.84)	0.79
TG (mmol/L), M (Q₁, Q₃)	1.45 (1.02 - 1.76)	1.05 (0.86 - 1.35)	<0.01	2.02 (1.15 - 2.16)	1.44 (0.99 - 2.45)	0.49	0.94 (0.80 - 1.05)	1.36 (1.08 - 2.13)	0.02
**Tumor Biomarkers**									
CEA (ng/ml), M (Q₁, Q₃)	2.05 (1.47 - 3.12)	2.61 (1.61 - 3.46)	0.19	1.91 (1.64 - 2.68)	2.28 (1.35 - 2.82)	0.91	3.40 (2.51 - 4.02)	3.20 (2.45 - 5.60)	0.70
SCC-Ag (ng/ml), M (Q₁, Q₃)	0.90 (0.70 - 1.18)	1.60 (1.02 - 2.04)	<0.01	0.93 (0.87 - 1.28)	1.26 (0.81 - 1.71)	0.27	0.80 (0.60 - 1.08)	1.00 (0.60 - 1.68)	0.43
CA-199 (U/ml), M (Q₁, Q₃)	8.79 (6.13 - 12.03)	9.36 (6.08 - 14.20)	0.51	8.75 (4.19 - 19.80)	9.84 (6.14 - 16.52)	0.59	3.66 (2.61 - 6.39)	6.25 (4.74 - 12.28)	0.02
CA-125 (U/ml), M (Q₁, Q₃)	7.08 (4.55 - 9.83)	9.77 (6.55 - 12.40)	0.01	10.53 (3.84 - 14.10)	8.78 (6.20 - 12.23)	0.99	8.50 (6.15 - 11.57)	9.39 (7.34 - 12.00)	0.69
CA-724 (U/ml), M (Q₁, Q₃)	2.09 (1.73 - 4.86)	3.80 (2.26 - 6.35)	0.03	2.32 (1.72 - 2.77)	3.48 (2.60 - 6.64)	0.06	2.81 (1.53 - 5.45)	3.04 (1.79 - 5.93)	0.71
**Tumor-related characteristics**									
Location, n (%)			0.83			0.99			0.12
Upper thoracic segment	2 (5.00)	2 (3.08)		0 (0.00)	0 (0.00)		0 (0.00)	2 (7.14)	
Middle thoracic segment	19 (47.50)	29 (44.62)		3 (33.33)	10 (27.78)		7 (70.00)	9 (32.14)	
Lower thoracic segment	19 (47.50)	34 (52.31)		6 (66.67)	26 (72.22)		3 (30.00)	17 (60.71)	
Histology, n (%)			0.11			0.23			0.55
Adenocarcinoma	1 (2.50)	9 (13.85)		0 (0.00)	4 (11.11)		0 (0.00)	3 (10.71)	
Squamous carcinoma	39 (97.50)	56 (86.15)		9 (100.00)	32 (88.89)		10 (100.00)	25 (89.29)	
**Treatment plan, n (%)**			<0.01			0.80			0.06
NC	18 (45.00)	47 (72.31)		6 (66.67)	28 (77.78)		1 (10.00)	14 (50.00)	
NC+ immunotherapy	22 (55.00)	18 (27.69)		3 (33.33)	8 (22.22)		9 (90.00)	14 (50.00)	

*TRG* Tumor regression grade, *BMI* Body mass index, *PFT* Pulmonary function tests, *dNLR* derived neutrophils/ (leukocytes minus neutrophils) ratio, *NLR* Neutrophil–lymphocyte ratio, *PLR* Platelet–lymphocyte ratio, *LMR* Lymphocyte-to-monocyte ratio, *SII* Systemic immune-inflammation index, *TBil* Total bilirubin, *ALT* Alanine aminotransferase, *AST* Aspartate aminotransferase, *LDH* Lactate dehydrogenase, *Cr* Creatinine, *UA* Uric acid, *Glu* Glucose, *TP* Total protein, *ALB* Albumin, *PA* Prealbumin, *PNI* Prognostic nutritional index, *Cho* Cholesterol, *HDL* High-density lipoprotein, *LDL* Low-density lipoprotein, *TG* Triglyceride, *CEA* Carcinoembryonic antigen, *SCC-Ag* Squamous cell carcinoma antigen, *CA-199* Carbohydrate antigen 199, *CA-125* Carbohydrate antigen 125, *CA-724* Carbohydrate antigen 724, *NC* Neoadjuvant chemotherapy

**Table 3:** Preoperative clinical characteristics of patients with ypTNM stage I and II-IVA in the training and validation cohorts (internal and external)

**Characteristics**	**Training Cohort**			**Internal Validation Cohort**			**External Validation Cohort**		
	**Stage I (*n*=48)**	**Stage II-IVA (*n*=57)**	***P* value**	**Stage I (*n*=12)**	**Stage II-IVA (*n*=33)**	***P* value**	**Stage I (*n*=10)**	**Stage II-IVA (*n*=28)**	***P* value**
**Demographics**									
Age (years), M (Q₁, Q₃)	63.50 (59.00 - 66.25)	61.00 (57.00 - 68.00)	0.92	65.00 (61.50 - 70.25)	63.00 (58.00 - 66.00)	0.263	66.00 (63.25 - 70.00)	62.00 (57.00 - 70.25)	0.21
Gender, n(%)			0.71			0.86			0.16
Female	8 (16.67)	8 (14.04)		2 (16.67)	3 (9.09)		2 (20.00)	1 (3.57)	
Male	40 (83.33)	49 (85.96)		10 (83.33)	30 (90.91)		8 (80.00)	27 (96.43)	
BMI (kg/m2), M (Q₁, Q₃)	22.23 (21.41 - 23.69)	22.47 (20.42 - 23.55)	0.39	26.98 (26.73 - 28.14)	26.89 (26.20 - 28.31)	0.61	21.89 (20.62 - 24.00)	22.77 (21.04 - 24.57)	0.61
Smoking history, n(%)			0.30			0.87			0.27
No	18 (37.50)	16 (28.07)		5 (41.67)	11 (33.33)		2 (20.00)	12 (42.86)	
Yes	30 (62.50)	41 (71.93)		7 (58.33)	22 (66.67)		8 (80.00)	16 (57.14)	
Alcohol use, n (%)			0.99			0.59			0.26
No	16 (33.33)	19 (33.33)		2 (16.67)	10 (30.30)		2 (20.00)	13 (46.43)	
Yes	32 (66.67)	38 (66.67)		10 (83.33)	23 (69.70)		8 (80.00)	15 (53.57)	
Family history of tumors, n (%)			0.99			0.17			0.99
No	46 (95.83)	55 (96.49)		10 (83.33)	32 (96.97)		8 (80.00)	22 (78.57)	
Yes	2 (4.17)	2 (3.51)		2 (16.67)	1 (3.03)		2 (20.00)	6 (21.43)	
PFT, N(%)			0.05			0.11			0.99
Normal	32 (66.67)	27 (47.37)		9 (75.00)	16 (48.48)		6 (60.00)	15 (53.57)	
Abnormal	16 (33.33)	30 (52.63)		3 (25.00)	17 (51.52)		4 (40.00)	13 (46.43)	
**Complete blood count**									
Hb (g/L), M (Q₁, Q₃)	129.00 (120.00 - 136.00)	128.00 (116.00 - 138.00)	0.76	130.00 (118.50 - 135.00)	133.00 (120.00 - 141.00)	0.26	127.00 (111.00 - 132.00)	131.00 (122.75 - 137.00)	0.14
dNLR, M (Q₁, Q₃)	1.50 (1.20 - 1.92)	1.30 (1.05 - 1.88)	0.28	1.61 (1.21 - 2.10)	1.23 (1.01 - 1.68)	0.23	1.84 (1.41 - 2.42)	1.59 (1.15 - 2.28)	0.39
NLR, M (Q₁, Q₃)	2.08 (1.54 - 2.70)	1.97 (1.40 - 2.49)	0.43	2.18 (1.61 - 2.87)	1.81 (1.38 - 2.45)	0.27	3.13 (2.00 - 3.29)	2.20 (1.81 - 3.44)	0.52
PLR, M (Q₁, Q₃)	129.49 (102.69 - 160.03)	133.96 (114.44 - 174.02)	0.57	142.54 (100.27 - 185.28)	142.27 (102.69 - 182.86)	0.97	125.10 (102.12 - 167.61)	160.28 (127.19 - 176.81)	0.29
LMR, M (Q₁, Q₃)	3.26 (2.54 - 3.82)	3.38 (2.49 - 4.27)	0.66	3.63 (2.51 - 3.96)	2.97 (2.12 - 4.60)	0.75	2.56 (2.13 - 3.14)	2.71 (2.40 - 3.48)	0.55
SII, M (Q₁, Q₃)	401.19 (284.45 - 584.13)	450.69 (292.57 - 592.36)	0.67	453.46 (295.22 - 716.08)	381.52 (242.54 - 578.73)	0.57	458.35 (409.08 - 673.47)	555.70 (281.08 - 871.69)	0.88
**Liver & renal function**									
TBil (μmol/L), M (Q₁, Q₃)	8.80 (7.35 - 10.43)	8.70 (7.30 - 12.10)	0.74	9.00 (7.50 - 11.83)	9.50 (7.60 - 13.00)	0.84	10.10 (7.80 - 12.32)	10.30 (8.75 - 12.90)	0.75
ALT (U/L), M (Q₁, Q₃)	17.00 (12.75 - 23.50)	16.00 (14.00 - 25.00)	0.95	13.00 (9.50 - 16.00)	15.00 (11.00 - 20.00)	0.37	23.50 (14.25 - 26.00)	15.50 (10.75 - 25.25)	0.25
AST (U/L), M (Q₁, Q₃)	19.00 (16.75 - 22.25)	18.00 (16.00 - 23.00)	0.77	16.00 (13.75 - 22.25)	18.00 (13.00 - 22.00)	0.80	22.00 (19.00 - 28.00)	16.50 (14.00 - 21.25)	0.04
LDH (U/L), M (Q₁, Q₃)	198.50 (176.00 - 211.50)	191.00 (171.00 - 213.00)	0.36	209.50 (185.25 - 232.25)	186.00 (166.00 - 212.00)	0.07	223.00 (210.00 - 232.00)	208.00 (173.50 - 241.25)	0.41
Urea (mmol/L), M (Q₁, Q₃)	5.47 (4.27 - 6.50)	5.70 (4.67 - 6.60)	0.45	5.00 (4.35 - 6.53)	5.10 (4.00 - 6.28)	0.97	5.70 (4.83 - 6.83)	5.10 (4.18 - 5.60)	0.12
Cr (μmol/L), M (Q₁, Q₃)	71.50 (62.00 - 80.00)	70.00 (63.00 - 80.00)	0.82	68.00 (64.50 - 76.25)	72.00 (66.00 - 80.00)	0.55	66.00 (57.00 - 75.50)	68.50 (60.00 - 76.00)	0.69
UA (μmol/L), M (Q₁, Q₃)	287.00 (248.25 - 361.25)	309.00 (262.00 - 352.00)	0.73	301.00 (278.00 - 340.75)	307.00 (271.00 - 370.00)	0.79	291.00 (209.50 - 340.25)	304.00 (254.75 - 348.75)	0.58
**Nutritional status**									
Glu (mmol/L), M (Q₁, Q₃)	5.28 (4.74 - 5.56)	5.10 (4.67 - 5.37)	0.20	5.23 (4.98 - 5.81)	5.21 (4.76 - 6.04)	0.99	5.14 (4.56 - 5.84)	5.12 (4.74 - 5.88)	0.86
TP (g/L), M (Q₁, Q₃)	67.45 (65.25 - 71.47)	68.10 (63.90 - 70.90)	0.99	69.05 (64.05 - 70.50)	66.70 (63.70 - 72.50)	0.92	68.00 (67.00 - 71.50)	64.70 (62.12 - 70.75)	0.10
ALB (g/L), M (Q₁, Q₃)	43.15 (40.65 - 44.65)	42.80 (40.40 - 45.60)	0.87	42.80 (41.40 - 43.68)	41.50 (40.70 - 44.00)	0.50	42.65 (41.12 - 45.25)	40.95 (39.22 - 42.75)	0.11
PA (g/L), M (Q₁, Q₃)	25.30 (22.90 - 26.52)	23.30 (18.70 - 26.40)	0.05	23.70 (20.82 - 28.68)	23.50 (20.00 - 27.10)	0.57	20.50 (19.72 - 21.80)	21.85 (19.45 - 25.72)	0.16
PNI, M (Q₁, Q₃)	50.85 (47.39 - 53.21)	51.10 (48.00 - 54.90)	0.44	51.08 (46.36 - 52.34)	48.85 (46.35 - 53.10)	0.98	48.50 (47.74 - 54.85)	48.45 (45.51 - 50.99)	0.23
**Lipid levels**									
Cho (mmol/L), M (Q₁, Q₃)	5.11 (4.44 - 5.70)	4.84 (4.26 - 5.47)	0.28	5.17 (4.22 - 5.81)	4.73 (4.23 - 5.29)	0.46	4.02 (3.65 - 5.01)	4.33 (4.04 - 4.83)	0.35
HDL (mmol/L), M (Q₁, Q₃)	1.22 (1.03 - 1.45)	1.25 (1.05 - 1.50)	0.55	1.18 (1.04 - 1.40)	1.10 (1.02 - 1.22)	0.28	1.17 (1.06 - 1.39)	1.11 (0.96 - 1.38)	0.36
LDL (mmol/L), M (Q₁, Q₃)	3.12 (2.58 - 3.76)	2.88 (2.42 - 3.41)	0.17	3.02 (2.85 - 3.78)	2.84 (2.37 - 3.49)	0.26	2.20 (1.96 - 2.64)	2.45 (2.08 - 2.84)	0.44
TG (mmol/L), M (Q₁, Q₃)	1.19 (0.98 - 1.54)	1.13 (0.86 - 1.48)	0.23	1.47 (0.99 - 2.23)	1.48 (1.08 - 2.49)	0.76	0.98 (0.80 - 1.29)	1.17 (1.00 - 2.13)	0.15
**Tumor Biomarkers**									
CEA (ng/ml), M (Q₁, Q₃)	2.00 (1.48 - 2.93)	2.93 (1.64 - 3.61)	0.05	1.67 (1.30 - 1.97)	2.66 (1.54 - 3.02)	0.03	2.69 (2.32 - 3.75)	3.62 (2.73 - 5.77)	0.10
SCC-Ag (ng/ml), M (Q₁, Q₃)	0.91 (0.74 - 1.27)	1.70 (1.10 - 2.10)	<0.01	1.02 (0.85 - 1.30)	1.31 (0.80 - 1.70)	0.30	0.80 (0.60 - 1.08)	1.00 (0.60 - 1.68)	0.46
CA-199 (U/ml), M (Q₁, Q₃)	8.44 (5.30 - 12.22)	9.45 (7.82 - 14.20)	0.11	5.80 (4.60 - 9.09)	11.20 (7.68 - 18.00)	0.02	3.28 (2.61 - 6.19)	6.25 (4.82 - 12.28)	0.01
CA-125 (U/ml), M (Q₁, Q₃)	7.57 (5.12 - 11.22)	9.18 (6.55 - 11.70)	0.11	4.86 (3.83 - 7.37)	9.66 (7.35 - 13.33)	<0.01	7.07 (5.52 - 11.00)	9.60 (7.39 - 12.00)	0.22
CA-724 (U/ml), M (Q₁, Q₃)	2.23 (1.80 - 4.71)	3.85 (2.14 - 7.33)	0.03	2.31 (1.68 - 2.83)	3.91 (2.85 - 6.87)	0.01	2.04 (1.46 - 4.39)	3.20 (1.94 - 5.93)	0.27
**Tumor-related characteristics**									
Location, n (%)			0.21			0.98			0.12
Upper thoracic segment	3 (6.25)	1 (1.75)		0 (0.00)	0 (0.00)		0 (0.00)	2 (7.14)	
Middle thoracic segment	24 (50.00)	24 (42.11)		4 (33.33)	9 (27.27)		7 (70.00)	9 (32.14)	
Lower thoracic segment	21 (43.75)	32 (56.14)		8 (66.67)	24 (72.73)		3 (30.00)	17 (60.71)	
Histology, n (%)			0.17			0.45			0.55
Adenocarcinoma	2 (4.17)	8 (14.04)		1 (8.33)	8 (24.24)		0 (0.00)	3 (10.71)	
Squamous carcinoma	46 (95.83)	49 (85.96)		11 (91.67)	25 (75.76)		10 (100.00)	25 (89.29)	
**Treatment plan, n (%)**			0.60			0.99			0.06
NC	31 (64.58)	34 (59.65)		9 (75.00)	25 (75.76)		1 (10.00)	14 (50.00)	
NC+ immunotherapy	17 (35.42)	23 (40.35)		3 (25.00)	8 (24.24)		9 (90.00)	14 (50.00)	

*BMI* Body mass index, *PFT* Pulmonary function tests, *dNLR* derived neutrophils/ (leukocytes minus neutrophils) ratio, *NLR* Neutrophil–lymphocyte ratio, *PLR* Platelet–lymphocyte ratio, *LMR* Lymphocyte-to-monocyte ratio, *SII* Systemic immune-inflammation index, *TBil* Total bilirubin, *ALT* Alanine aminotransferase, *AST* Aspartate aminotransferase, *LDH* Lactate dehydrogenase, *Cr* Creatinine, *UA* Uric acid, *Glu* Glucose, *TP* Total protein, *ALB* Albumin, *PA* Prealbumin, *PNI* Prognostic nutritional index, *Cho* Cholesterol, *HDL* High-density lipoprotein, *LDL* Low-density lipoprotein, *TG* Triglyceride, *CEA* Carcinoembryonic antigen, *SCC-Ag* Squamous cell carcinoma antigen, *CA-199* Carbohydrate antigen 199, *CA-125* Carbohydrate antigen 125, *CA-724* Carbohydrate antigen 724, *NC* Neoadjuvant chemotherapy

### Kaplan-Meier Survival Analysis

**Fig. 2:**
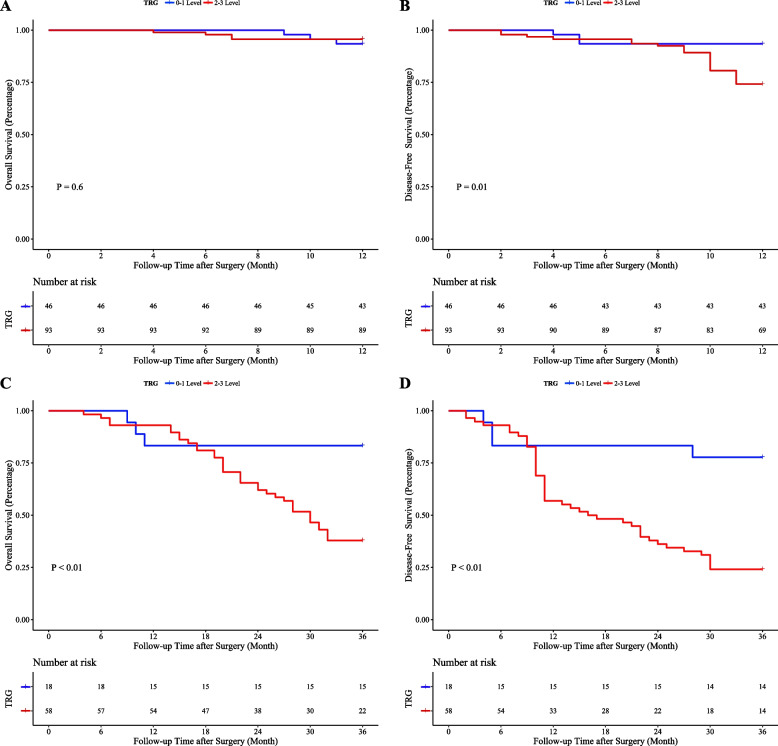
Kaplan-Meier survival curves for patients graded according to TRG. **A** 1-year overall survival; (**B**) 1-year disease-free survival; (**C**) 3-year overall survival; (**D**) 3-year disease-free survival. TRG: tumor regression grade

A total of 150 patients from Qilu Hospital were included in this study, with 11 lost to follow-up. The follow-up period for the remaining 139 patients ranged from 4.0 to 36.0 months. The 1-year rates of DFS and OS were 80.6% and 95.0%, respectively, while the 3-year rates were 36.8% and 48.7%. Patients in the TRG grade 0-1 group showed no significant difference in 1-year rates of OS compared to those in the TRG grade 2-3 group (93.5% vs. 95.7%, *P*=0.60). However, significant differences were observed in 1-year rates of DFS (93.5% vs. 74.2%, P=0.01), 3-year rates of OS (83.3% vs. 37.9%, *P*<0.01), and 3-year rates of DFS (77.8% vs. 24.1%, *P*<0.01) (Fig. 2A-D).

**Fig. 3:**
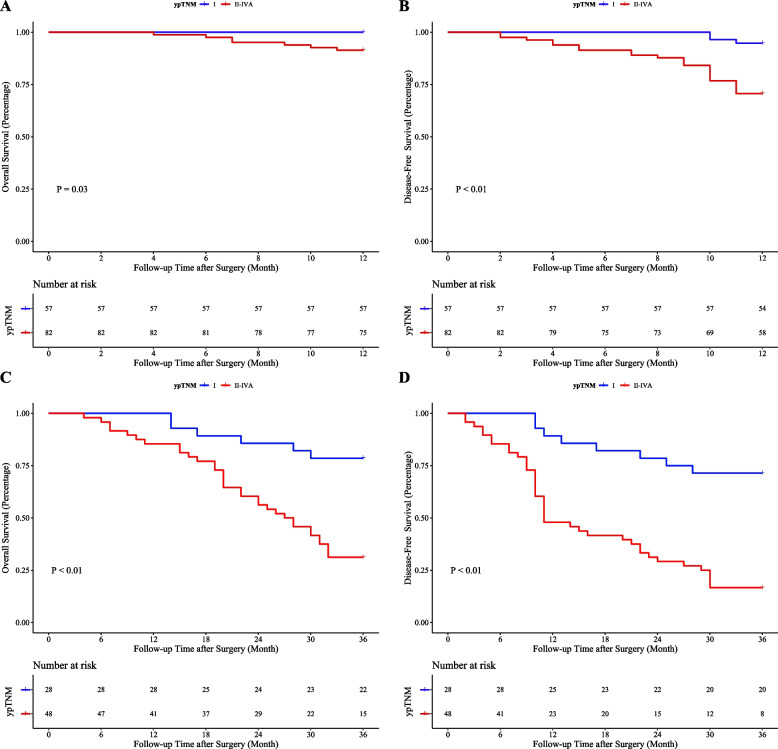
Kaplan-Meier survival curves for patients graded according to ypTNM. **A** 1-year overall survival; (**B**) 1-year disease-free survival; (**C**) 3-year overall survival; (**D**) 3-year disease-free survival

**Fig. 4:**
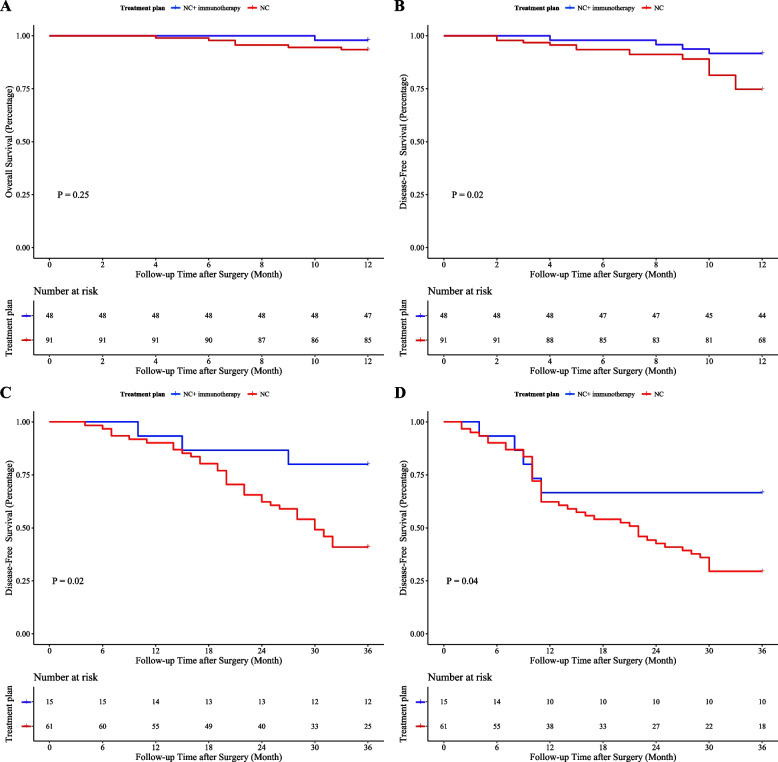
Kaplan-Meier survival curves based on the patient's neoadjuvant treatment plan. **A** 1-year overall survival; (**B**) 1-year disease-free survival; (**C**) 3-year overall survival; (**D**) 3-year disease-free survival. NC: neoadjuvant chemotherapy

As shown in Fig. 3A-D, in the ypTNM classification, the 1-year survival rate of stage I patients was significantly better than that of stage II-IVA patients (OS: 100.0% vs. 91.5%, *P*=0.03; DFS: 94.7% vs. 70.7%, *P*<0.01). The 3-year outcomes also showed significant advantages for Stage I patients in both OS (78.6% vs. 31.3%, *P*<0.01) and DFS (71.4% vs. 16.7%, *P*<0.01). The addition of immunotherapy to neoadjuvant chemotherapy (NC) did not significantly affect the 1-year OS compared to NC alone (97.9% vs. 93.4%, P=0.25). However, significant improvements were noted in 1-year DFS (91.7% vs. 74.7%, *P*=0.02), 3-year OS (80.0% vs. 41.0%, *P*=0.02), and 3-year DFS (66.7% vs. 29.5%, *P*<0.01) for patients receiving the combined treatment (Fig. 4A-D).

### Factors associated with TRG grade and ypTNM stage

**Fig. 5:**
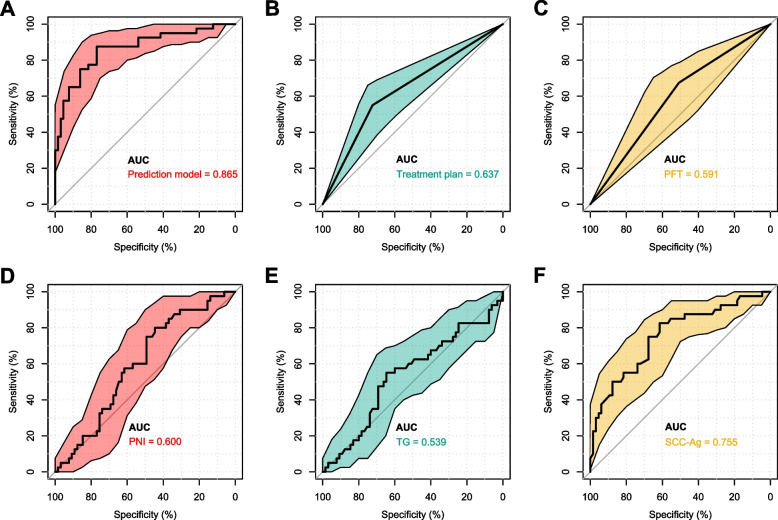
ROC curves and area under the curve for risk factors independently associated with TRG level in patients with esophageal cancer after neoadjuvant therapy. ROC: receiver operating characteristic; AUC: area under the ROC curve; PFT: pulmonary function tests; PNI: prognostic nutritional index; TG: triglyceride; SCC-Ag: squamous cell carcinoma antigen

**Fig. 6:**
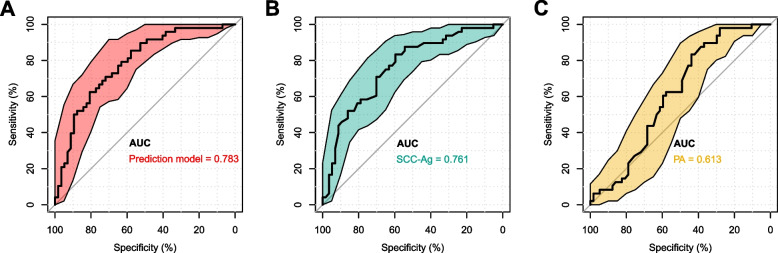
ROC curves and area under the curve for risk factors independently associated with ypTNM stage in patients with esophageal cancer after neoadjuvant therapy. ROC: receiver operating characteristic; AUC: area under the ROC curve; SCC-Ag: squamous cell carcinoma antigen; PA: prealbumin

Both univariate and multivariate logistic regression analyses were performed in the training cohort to determine the variables which affect the TRG grade and ypTNM stage. The results of these analyses are provided in Table 4. The univariate logistic regression analysis revealed that factors such as age, family history, pulmonary function tests (PFT), hemoglobin levels (Hb), prognostic nutritional index (PNI), high-density lipoprotein (HDL), triglyceride (TG), squamous cell carcinoma antigen (SCC-Ag), carbohydrate antigen 125 (CA-125), the histologic type of EC, and the inclusion of immunotherapy significantly affected the TRG stage post-NT (P value < 0.20). Subsequent multivariate logistic regression analysis using forward stepwise regression identified several independent predictors of TRG level 0-1: PFT [Odd Ratio (OR) = 0.33; 95% confidence interval (CI): 0.11-0.99; *P* < 0.05], PNI (OR = 0.90; 95% CI: 0.81-0.99; *P* < 0.05), TG levels (OR = 3.58; 95% CI: 1.25-10.22; *P* = 0.02), serum SCC-Ag levels (OR = 0.14; 95% CI: 0.05-0.40; *P* < 0.01) and the inclusion of immunotherapy (OR = 6.49; 95% CI: 2.07-20.36; *P* < 0.01). Additionally, univariate analysis indicated that body mass index (BMI), PFT, glucose (Glu), prealbumin (PA), low-density lipoprotein (LDL), carcinoembryonic antigen (CEA), SCC-Ag, CA-125 levels, the histologic type of EC, and the inclusion of immunotherapy were correlated with ypTNM stage post-NT (*P* value < 0.20). Further, multivariate logistic regression analysis identified blood PA level (OR = 1.12; 95% CI: 1.00-1.25; *P* < 0.05) and serum SCC-Ag level (OR = 0.21; 95% CI: 0.08-0.52; *P* < 0.01) as independent predictors of ypTNM stage I. For some continuous variables (PFT, PNI, TG, PA, SCC-Ag), optimal cutoff values were determined through ROC curve analysis, with these values subsequently used to convert them into binary variables for inclusion in the regression analysis, as outlined in Figs. 5, 6, Supplementary Tables 1 and 2.

**Table 4:** Univariate and multivariate logistic regression analysis of independent factors for TRG level 0-1 and ypTNM stage I in the training cohort

**Variable**	**Univariate analysis (TRG)**		**Multivariate analysis (TRG)**		**Univariate analysis (ypTNM)**		**Multivariate analysis (ypTNM)**	
	**OR (95%CI)**	***P* value**	**OR (95%CI)**	***P* value**	**OR (95%CI)**	***P* value**	**OR (95%CI)**	***P* value**
**Demographics**								
Age (years), M (Q₁, Q₃)	0.95 (0.90, 1.01)	**0.09**			0.99 (0.95, 1.05)	0.90		
Gender, n(%)		0.96						
Female	Ref.				Ref.			
Male	1.03 (0.34, 3.09)				0.82 (0.28, 2.37)	0.71		
BMI (kg/m^2^), M (Q₁, Q₃)	1.05 (0.86, 1.29)	0.61			1.15 (0.94, 1.41)	**0.18**		
Smoking history, n(%)		0.65				0.31		
No	Ref.				Ref.			
Yes	0.83 (0.36, 1.91)				0.65 (0.29, 1.48)			
Alcohol use, n (%)		0.78				0.99		
No	Ref.				Ref.			
Yes	0.89 (0.39, 2.04)				1.00 (0.44, 2.26)			
Family history of tumors, n (%)		**0.16**				0.86		
No	Ref.				Ref.			
Yes	5.19 (0.52, 51.71)				1.20 (0.16, 8.82)			
PFT, N(%)		**0.07**		**<0.05**		**0.05**		
Normal	Ref.		Ref.		Ref.			
Abnormal	0.47 (0.21, 1.06)		0.33 (0.11, 0.99)		0.45 (0.20, 1.00)			
**Complete blood count**								
Hb (g/L), M (Q₁, Q₃)	0.98 (0.95, 1.01)	**0.12**			1.01 (0.99, 1.04)	0.35		
dNLR, M (Q₁, Q₃)	1.06 (0.79, 1.43)	0.71			0.98 (0.72, 1.32)	0.89		
NLR, M (Q₁, Q₃)	1.05 (0.91, 1.21)	0.52			1.01 (0.88, 1.17)	0.88		
PLR, M (Q₁, Q₃)	1.00 (0.99, 1.01)	0.49			1.00 (0.99, 1.01)	0.98		
LMR, M (Q₁, Q₃)	0.87 (0.65, 1.17)	0.35			0.89 (0.67, 1.18)	0.41		
SII, M (Q₁, Q₃)	1.00 (0.99, 1.01)	0.99			1.00 (0.99, 1.00)	0.72		
**Liver & renal function**								
TBil (μmol/L), M (Q₁, Q₃)	0.94 (0.85, 1.04)	0.22			0.98 (0.90, 1.07)	0.67		
ALT (U/L), M (Q₁, Q₃)	1.01 (0.98, 1.04)	0.68			0.99 (0.96, 1.02)	0.48		
AST (U/L), M (Q₁, Q₃)	1.01 (0.97, 1.06)	0.55			0.99 (0.95, 1.04)	0.69		
LDH (U/L), M (Q₁, Q₃)	1.01 (0.99, 1.01)	0.21			1.00 (0.99, 1.01)	0.43		
Urea (mmol/L), M (Q₁, Q₃)	0.92 (0.75, 1,13)	0.43			0.90 (0.73, 1.12)	0.36		
Cr (μmol/L), M (Q₁, Q₃)	0.99 (0.97, 1.02)	0.81			1.00 (0.98, 1.02)	0.99		
UA (μmol/L), M (Q₁, Q₃)	1.00 (0.99, 1.01)	0.92			0.99 (0.99, 1.00)	0.82		
**Nutritional status**								
Glu (mmol/L), M (Q₁, Q₃)	1.20 (0.81, 1.77)	0.36			1.52 (0.97, 2.40)	**0.07**		
TP (g/L), M (Q₁, Q₃)	0.99 (0.93, 1.07)	0.95			1.02 (0.95, 1.09)	0.67		
ALB (g/L), M (Q₁, Q₃)	0.99 (0.89, 1.10)	0.85			1.02 (0.92, 1.13)	0.69		
PA (g/L), M (Q₁, Q₃)	1.04 (0.96, 1.14)	0.31			1.10 (1.01, 1.20)	**0.03**	1.12 (1.00, 1.25)	**<0.05**
PNI, M (Q₁, Q₃)	0.95 (0.88, 1.03)	**0.19**	0.90 (0.81, 0.99)	**<0.05**	0.98 (0.91, 1.06)	0.65		
**Lipid levels**								
Cho (mmol/L), M (Q₁, Q₃)	1.21 (0.81, 1.82)	0.36			1.27 (0.85, 1.90)	0.25		
HDL (mmol/L), M (Q₁, Q₃)	0.38 (0.09, 1.53)	**0.17**			0.66 (0.18, 2.48)	0.53		
LDL (mmol/L), M (Q₁, Q₃)	1.32 (0.78, 2.22)	0.31			1.54 (0.91, 2.61)	**0.11**		
TG (mmol/L), M (Q₁, Q₃)	3.33 (1.44, 7.74)	**<0.01**	3.58 (1.25, 10.22)	**0.02**	1.28 (0.67, 2.42)	0.45		
**Tumor Biomarkers**								
CEA (ng/ml), M (Q₁, Q₃)	0.82 (0.60, 1.12)	0.21			0.75 (0.55, 1.02)	**0.06**		
SCC -Ag (ng/ml), M (Q₁, Q₃)	0.20 (0.09, 0.45)	**<0.01**	0.14 (0.05, 0.40)	**<0.01**	0.19 (0.09, 0.42)	**<0.01**	0.21 (0.08, 0.52)	**<0.01**
CA-199 (U/ml), M (Q₁, Q₃)	0.99 (0.95, 1.03)	0.56			1.01 (0.99, 1.02)	0.48		
CA-125 (U/ml), M (Q₁, Q₃)	0.87 (0.78, 0.97)	**0.01**			0.92 (0.84, 1.02)	**0.11**		
CA-724 (U/ml), M (Q₁, Q₃)	0.99 (0.94, 1.05)	0.82			0.99 (0.95, 1.05)	0.83		
**Tumor-related characteristics**								
Location, n (%)		0.82				0.31		
Upper thoracic segment	Ref.				Ref.			
Middle thoracic segment	0.66 (0.09, 5.06)	0.69			0.33 (0.03, 3.44)			
Lower thoracic segment	0.56 (0.08, 4.29)	0.58			0.22 (0.02, 2.25)			
Histology, n (%)		**0.09**				**0.11**		
Adenocarcinoma	Ref.				Ref.			
Squamous carcinoma	6.27 (0.76, 51.49)				3.76 (0.76, 18,62)			
**Treatment plan, n (%)**		**<0.01**		**<0.01**		0.60		
NC	Ref.		Ref.		Ref.			
NC+ immunotherapy	3.19 (1.40, 7.29)		6.49 (2.07, 20.36)		0.81 (0.37, 1.79)			

*TRG* Tumor regression grade, *OR* Odds ratio, *CI* Confidence interval, *BMI* Body mass index, *PFT* Pulmonary function tests, *dNLR* derived neutrophils/ (leukocytes minus neutrophils) ratio, *NLR* Neutrophil–lymphocyte ratio, *PLR* Platelet–lymphocyte ratio, *LMR* Lymphocyte-to-monocyte ratio, *SII* Systemic immune-inflammation index, *TBil* Total bilirubin, *ALT* Alanine aminotransferase, *AST* Aspartate aminotransferase, *LDH* Lactate dehydrogenase, *Cr* Creatinine, *UA* Uric acid, *Glu* Glucose, *TP* Total protein, *ALB* Albumin, *PA* Prealbumin, *PNI* Prognostic nutritional index, *Cho* Cholesterol, *HDL* High-density lipoprotein, *LDL* Low-density lipoprotein *TG* Triglyceride, *CEA* Carcinoembryonic antigen, *SCC-Ag* Squamous cell carcinoma antigen, *CA-199* Carbohydrate antigen 199, *CA-125* Carbohydrate antigen 125, *CA-724* Carbohydrate antigen 724, *NC* Neoadjuvant chemotherapy

### Nomogram construction

**Fig. 7:**
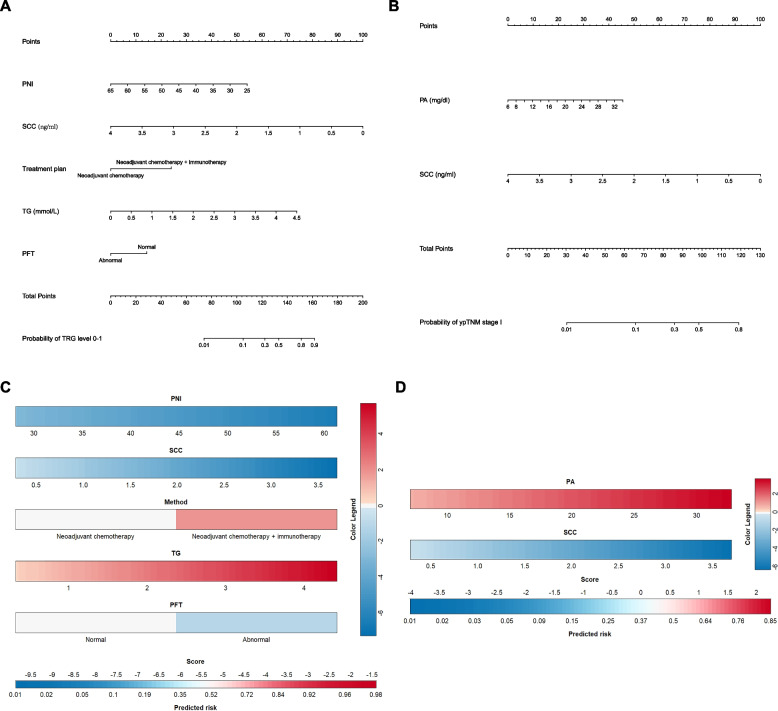
Nomograms and standard color cards for predicting TRG level and ypTNM stage in patients with esophageal cancer after neoadjuvant therapy. **A** TRG level nomogram; (**B**) ypTNM stage nomogram; (**C**) TRG level standard color card; (**D**) ypTNM stage standard color card. TRG: tumor regression grade; ypTNM: neoadjuvant pathologic TNM; PFT: pulmonary function tests; PNI: prognostic nutritional index; TG: triglyceride; SCC-Ag: squamous cell carcinoma antigen; PA: prealbumin

The logistic regression model identified five independent predictors for TRG level 0-1. A predictive nomogram for TRG level 0-1, derived from the coefficients of the logistic regression model, was plotted using the "rms" package in R statistical software (Fig. 7A). This nomogram comprises eight axes, with axes 2-6 representing the five identified variables. Scores for each variable are projected onto the top scale axis, and the total score is obtained by summing these individual scores. By aligning the total score with the lower total score axis, the probability of achieving TRG level 0-1 in NT patients can be estimated. Additionally, a logistic regression model correlating with ypTNM stage was developed (Fig. 7B), incorporating two independent predictors of ypTNM stage I. This nomogram includes five axes, where axes 2 and 3 correspond to the two predictive variables. The likelihood of ypTNM stage I in patients undergoing NT is predicted by summing the scores for each factor and aligning the total score with the lower total scale axis. Furthermore, we constructed nomograms' colorimetric cards (Fig. 7C and D), with blue representing risk factors and red representing protective factors, which makes the prediction model more intuitive.

### Predictive performance and nomogram validation

**Fig. 8:**
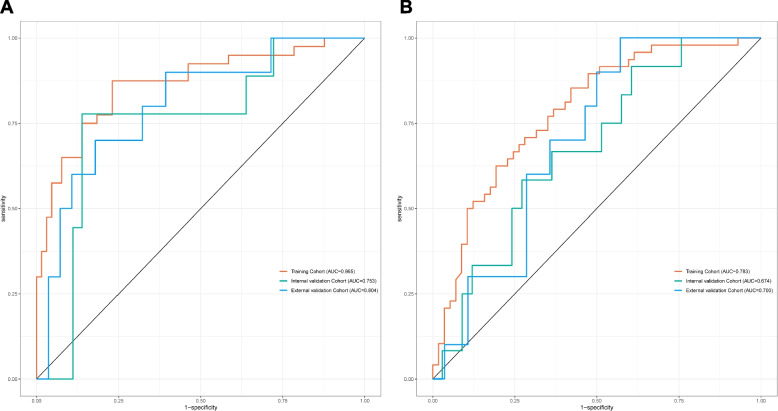
ROC curves of the nomogram used to predict the probability of TRG level and ypTNM stage in patients with esophageal cancer after neoadjuvant therapy in training and validation cohorts. **A** TRG level; (**B**) ypTNM stage. ROC: receiver operating characteristic; AUC: area under the ROC curve

For the TRG0-1 level prediction model, the discriminative ability was evaluated using ROC curves. Figure 8A illustrates the area under the ROC curve (AUC) for the training, internal validation, and external validation cohorts as 0.87 (95% CI: 0.79-0.94), 0.75 (95% CI: 0.57-0.93), and 0.80 (95% CI: 0.65-0.96), respectively, indicating satisfactory prediction accuracy of the nomogram. The optimal cut-off value for predicting TRG level 0-1 was approximately 64.42%, with a sensitivity of 0.88 and specificity of 0.77 **(**Supplementary Table 1**)**. The Hosmer-Lemeshow test indicated excellent calibration across the cohorts, with p-values of 0.35, 0.25, and 0.36 in the training, internal validation, and external validation groups, respectively. Calibration curves for the nomogram's predicted probabilities of TRG levels 0-1 showed strong concordance between predicted and observed outcomes (Supplementary Figures 1A, 1B, 1C).

When evaluating the effectiveness of NT using ypTNM stage, the AUCs for the training, internal validation, and external validation cohorts were 0.78 (95% CI: 0.70-0.87), 0.67 (95% CI: 0.51-0.84), and 0.70 (95% CI: 0.53-0.87), respectively, suggesting better predictive accuracy of the nomogram for ypTNM stage I (Fig. 8B). The optimal cut-off value for ypTNM stage I prediction was around 43.31%, with a sensitivity of 0.85 and specificity of 0.58 (Supplementary Table 2). The Hosmer-Lemeshow test demonstrated excellent calibration in all groups, with *P*-values of 0.86, 0.67, and 0.17 for the training, internal validation, and external validation groups, respectively. This affirmed the strong agreement between the predicted and actual results in the ypTNM stage I prediction model (Supplementary Figures 1D, 1E, 1F).

### Predictive nomogram’s clinical utility

**Fig. 9:**
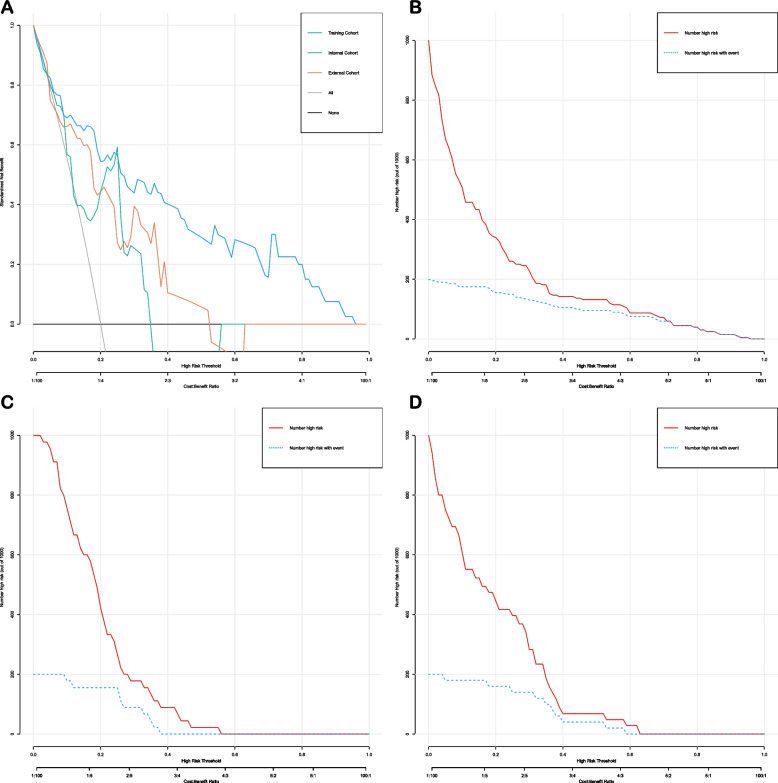
Decision curve analysis and clinical impact curves for the TRG level nomogram in the training and validation cohorts. **A** decision curve analysis; (**B**) clinical impact curves for training cohorts; (**C**) clinical impact curves for internal validation cohorts; (**D**) clinical impact curves for exteral validation cohorts

**Fig. 10:**
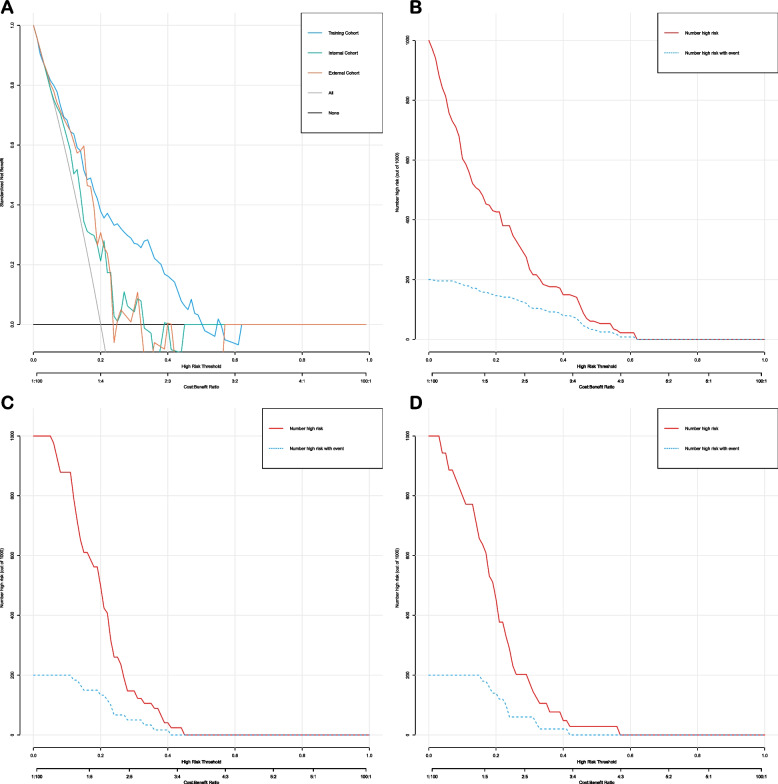
Decision curve analysis and clinical impact curves for the ypTNM stage nomogram in the training and validation cohorts. **A** decision curve analysis; **B** clinical impact curves for training cohorts; (**C**) clinical impact curves for internal validation cohorts; (**D**) clinical impact curves for exteral validation cohorts

The clinical utility of nomograms was evaluated through Decision Curve Analysis (DCA) and Clinical Impact Curves (CIC). For the TRG levels 0-1 prediction model, both DCA and CIC curves indicated a substantial net benefit for patients across a broad range of threshold probabilities (Fig. 9). Similarly, for ypTNM stage I, the model's DCA and CIC curves demonstrated significant net benefits within the 20-50% threshold range (Fig. 10).

## Discussion

EC, a notable health issue, is frequently detected in its latter stages and linked to a bleak outlook. The combination of neoadjuvant chemotherapy and immunotherapy shows great potential as a therapeutic strategy. Nevertheless, precisely forecasting its effectiveness continues to be a difficult task. Novel pathologic grading systems, such as ypTNM stage and TRG grade, provide superior predictive accuracy in EC patients who undergo preoperative NT compared to the conventional pTNM stage. Our survival analysis also suggests that patients with TRG grade 0-1 and ypTNM stage I also have better outcomes. Therefore, our study aimed to uncover factors that might predict the effectiveness of NT through these new pathologic grading systems. We created prognostic models for TRG grade 0-1 and ypTNM stage I by employing clinical features and laboratory testing. In addition, we evaluated the efficacy of the nomogram, which might serve as a useful tool for customizing treatment approaches in patients with locally progressed EC.

The results of the survival analysis of patients at Qilu Hospital show that for patients with TRG level 0-1, there was no difference in the 1-year OS, but both the DFS and the three-year OS and DFS were better than for patients with level 2-3. This suggests that patients who receive NT and achieve pathological and primary remission can significantly improve their Short-term DFS and long-term OS and DFS. Additionally, patients at ypTNM stage I have better short-term and long-term DFS and OS than those at stages II-IVA. This indicates that locally advanced EC patients who achieve a downgrade in pathological staging after NT are likely to have a better outcome. Therefore, using TRG grades and ypTNM classification can better predict the outcomes for patients undergoing NT, and accurately predicting a patient's TRG grade and ypTNM stage before surgery can effectively help clinicians estimate the effects of NT and choose the appropriate treatment plan. Finally, our analysis of NT modalities also included a group analysis, which showed that combining immunotherapy with NC significantly lowers recurrence rates and improves long-term prognostic outcomes compared to NC alone.

This study encompassed 150 EC patients from Qilu Hospital of Shandong University. To enhance the robustness of the analysis, these patients were randomized into training and validation groups. Additionally, 38 EC patients from Qianfoshan Hospital in the Shandong Province were incorporated as an external validation group, providing a diverse sample for the study. The absence of significant differences in variables between cohorts bolsters the reliability and generalizability of the study's results.

This study identified several factors impacting the TRG grade and ypTNM stage using both univariate and multivariate logistic regression analyses. In terms of TRG, PFT, PNI, TG levels, SCC-Ag levels, and combination immunotherapy emerged as independent predictors of TRG grade 0-1. For ypTNM stage, both PA and SCC-Ag levels were determined to be independent predictors of ypTNM stage I.

In terms of pulmonary function, it is generally accepted that NT leads to a decrease in a patient's pulmonary function [28–30]. However, our study results showed that patients with normal preoperative PFTs were more likely to achieve pCR than those with abnormal pulmonary function (OR = 0.33; 95% CI: 0.11-0.99; P < 0.05). A study by Katrien et al. indicated that in non-small cell lung cancer patients, a lower TRG was associated with improved pulmonary function, likely because a reduction in tumor size reduces airway compression [31]. It is well-known that EC patients with tumors near the trachea often experience airway invasion, esophageal respiratory fistula, airway compression, and other symptoms, leading to decreased pulmonary function [32–34]. Thus, although NT generally decreases pulmonary function, patients with normal PFT results may tend more toward a lower TRG.

Nutritional status plays a pivotal role in determining the effectiveness of neoadjuvant therapy. Several biomarkers, reflecting nutritional status, such as albumin to fibrinogen ratio, prealbumin to fibrinogen ratio, and nutritional risk index, have been employed to predict outcomes of neoadjuvant therapy [35, 36]. Our study found that serum PA level serves as an independent predictor of ypTNM staging post- NT. Research has demonstrated that in elderly patients with locally advanced esophageal squamous cell carcinoma, those with low prealbumin levels prior to neoadjuvant chemotherapy exhibit significantly lower OS compared to patients with high prealbumin levels. This observation aligns with the results of our study [37].This correlation might be attributed, in part, to the reduction of tumor burden leading to symptom improvement, such as alleviating dysphagia, which, in turn, enhances patient nutrition. Additionally, PA's strong association with systemic inflammatory response has been documented [38]. Future clinical trials are essential to further explore the causal link between serum prealbumin levels and the efficacy of neoadjuvant therapy.

The PNI, originally devised by Onodera et al., evaluates the nutritional and immunological status of patients undergoing gastrointestinal surgery [39]. It is derived from the serum albumin concentration and total lymphocyte count of a patient. Numerous subsequent studies have corroborated its association with the prognosis in various cancers. Several studies have evaluated PNI for predicting clinical outcomes in patients with esophageal cancer receiving NT [40–42]. Consistent with prior research, our findings confirm that PNI is an independent predictor for TRG after NT in patients with EC, and those with a higher PNI may be more sensitive to NT. These findings align with our experimental results, underscoring the significance of nutritional status in predicting the response to neoadjuvant therapy in patients with locally advanced EC.

Recent studies have established that lipid metabolism significantly contributes to tumor development and plays a role in drug resistance within tumors [43–46]. However, the specific relationship between serum lipidomics and tumors has been less explored. Notably, chemotherapeutic agents, particularly paclitaxel-based and platinum-based treatments, have been shown to influence serum lipid profiles by elevating TG levels at the conclusion of therapy [47, 48]. This may be attributed to the impact of chemotherapeutic drugs on lipid metabolism in liver cells [49].It has been reported that TG levels in serum are associated with susceptibility to NT for breast cancer [50, 51]. Our study extends these findings by establishing serum TG levels as an independent predictor of TRG after NT in EC patients (OR = 3.58; 95% CI: 1.25-10.22; P = 0.02). This marks the first identification of serum TG levels as predictive biomarkers for NT responsiveness in EC, offering a novel avenue for biomarker discovery in this context.

Regarding tumor markers, our study examined a range of common markers associated with EC, such as CEA, SCC-Ag, carbohydrate antigen 199, CA-125, and carbohydrate antigen 724 [52–55]. Through univariate and multivariate logistic regression analyses, we found that serum SCC-Ag levels effectively predict both TRG grade (OR = 0.14; 95% CI: 0.05-0.40; P < 0.01) and ypTNM stage (OR = 0.21; 95% CI: 0.08-0.52; P < 0.01) following NT in EC patients. Okamura et al. initially reported that serum SCC-Ag levels were indicative of TNM stage post-NT in esophageal squamous carcinoma patients [56]. Recent research also indicates that overexpression of SCC-Ag is a significant factor in drug resistance in esophageal adenocarcinoma [57]. One study found that elevated serum levels of SCC-Ag in patients undergoing NT for EC served as an independent predictor of non-R0 resection [58]. This observation aligns with our findings, where lower serum levels of SCC-Ag are associated with improved pathological responsiveness to NT in EC patients.

Despite the long-term survival benefits of NT for patients with locally advanced EC, the increased surgical challenges and poor prognosis for non-pCR patients highlight the need for more refined treatment strategies. Programmed cell death protein-1 (PD-1), discovered in 1992, is an immune checkpoint negatively regulating immune responses, expressed in T, B, and NK cells. It has two ligands, programmed cell death 1 ligand 1 (PD-L1) and programmed cell death 1 ligand 2 (PD-L2), which function to suppress local immune responses [59]. Immune checkpoint inhibitors (ICIs) such as PD-1 and PD-L1 inhibitors enhance anti-tumor immunity by deregulating the negative regulation of the immune system. Our predictive model suggests that combining NT with immunotherapy may enhance TRG outcomes compared to NT alone. This is also supported by the results of several clinical trials, in which NT combined with immunotherapy was able to increase the R0 resection and pCR rates [60–63]. Currently, immunotherapy is advancing to first- and second-line treatment in locally advanced EC. However, issues like comprehensive assessment of immunotherapy, identification of predictive biomarkers, and optimization of combined NT and immunotherapy regimens remain unresolved. Addressing these challenges constitutes the focus of our future research.

Notably, our prediction model AUC for TRG 0-1 after NT was as high as 0.87, suggesting that better lung function, higher PNI, higher serum TG levels, lower SCC-Ag levels, and combination immunotherapy could increase the likelihood of pathological remission after NT in EC patients. The high accuracy of this model was further validated by impressive results in both the internal and external validation cohorts. To evaluate the clinical utility of nomogram, we employed DCA) and CIC. Both DCA and CIC showed that the model could provide significant net benefits to patients over a wide range of high thresholds (Figure 9). Regarding the predictive model for ypTNM stage I, despite its high AUC of 0.783, the model's specificity was only 0.58. This limitation might stem from the inclusion of only two factors in the model: serum prealbumin and SCC-Ag levels. Future research will focus on identifying additional markers to enhance the precision of our prediction model. Moreover, subsequent DCA and CIC analyses confirmed that the model achieved substantial net benefits at thresholds between 20-50% (Figure 10).

Those patients who were able to achieve a pCR after NT were able to benefit more from NT compared to those who did not respond. Recently, there has been a debate on whether surgery is necessary for patients who attain pCR. However, the primary challenge lies in accurately assessing the effectiveness of NT and determining the patient's pathologic grade before surgery. The clinical predictive nomogram we developed aids thoracic surgeons in making informed clinical decisions by evaluating ypTNM and TRG grades post-NT, using preoperative clinical characteristics and laboratory tests. We have also visualized this predictive model by nomogram and developed an operational interface for these graphs on a website (https://ec-and-neoadjuvant--write-jianhaoqiu-zhanzhang.shinyapps.io/DynNom_TRG/ and https://ec-and-neoadjuvant--write-jianhaoqiu-zhanzhang.shinyapps.io/DynNom_ypTNM/), significantly streamlining the computational process and enhancing the model's clinical utility. The model's accuracy and effectiveness have been substantiated through internal and external validation, as well as DCA and CIC.

This study has several limitations. As a retrospective analysis, it may not fully represent the general applicability of our predictive nomograms, and uncontrolled confounding factors might be present. Moreover, despite the internal and external validation of the prediction model, selection bias observed in the training cohort could influence the internal validation cohort. Consequently, multiple external validations across different centers are required to confirm the column line graph's utility in diverse populations. Additionally, our study did not incorporate other imaging data, such as PET-CT and upper gastrointestinal tractography. Future research should aim to develop multicenter clinical prediction models that integrate comprehensive patient baseline data, laboratory tests, and imaging information.

## Conclusion

Utilizing clinical characteristics and laboratory tests from EC patient post- NT treatment, we established nomograms for predicting ypTNM stage I and TRG grade 0-1, achieving notable predictive accuracy. This tool empowers thoracic surgeons to anticipate patient sensitivity to NT beforehand, thereby facilitating the provision of customized treatment plans and achieving precision in treatment strategies.

### Supplementary Information

Supplementary Material 1. 

Supplementary Material 2.

Supplementary file 3: Supplementary Figure 1.Calibration curves of the prediction TRG level and ypTNM stage nomograms in the training and validation cohorts. (A) TRG level nomogram calibration curves for training cohorts; (B) TRG level nomogram calibration curves for internal validation cohorts; (C) TRG level nomogram calibration curves for exteral validation cohorts; (D) ypTNM stage nomogram calibration curves for training cohorts; (E) ypTNM stage nomogram calibration curves for internal validation cohorts; (F) ypTNM stage nomogram calibration curves for exteral validation cohorts. nomogram in the training cohort (A), internal validation cohort (B) and external validation cohort (C).

## Data Availability

No datasets were generated or analysed during the current study.

## Abbreviations

AUC: Area under the ROC curve
AJCC: American Joint Committee on Cancer
BMI: Body mass index
CA-125: Carbohydrate antigen 125
CEA: Carcinoembryonic antigen
CI: Confidence interval
CIC: Clinical impact curves
CT: Computed tomography
DCA: Decision curve analysis
EC: Esophageal cancer
Glu: Glucose
Hb: Hemoglobin levels
HDL: High-density lipoprotein
ICIs: Immune checkpoint inhibitors
IQR: Interquartile ranges
LDL: Low-density lipoprotein
NC: Neoadjuvant chemotherapy
NT: Neoadjuvant therapy
OR: Odd ratio
PD-1: Programmed cell death protein-1
PD-L1: Programmed cell death 1 ligand 1
PD-L2: Programmed cell death 1 ligand 2
PA: Prealbumin
pCR: Pathologic complete remission
PET-CT: Positron emission tomography–computed tomography
PFT: Pulmonary function test
PNI: Prognostic nutritional index
ROC: Receiver operating characteristic
SCC-Ag: Squamous cell carcinoma antigen
SD: Standard deviation
TG: Triglyceride
TRG: Tumor regression grade
UICC: Union for International Cancer Control
ypTNM: neoadjuvant pathologic TNM
