# Supplementary figures and images for: Nomograms to predict tumor regression grade (TRG) and ypTNM staging in patients with locally advanced esophageal cancer receiving neoadjuvant therapy
Source: World J Surg Oncol. 2024 Jul 27;22:198. doi: 10.1186/s12957-024-03474-7 (PMC11282666; doi:10.1186/s12957-024-03474-7)

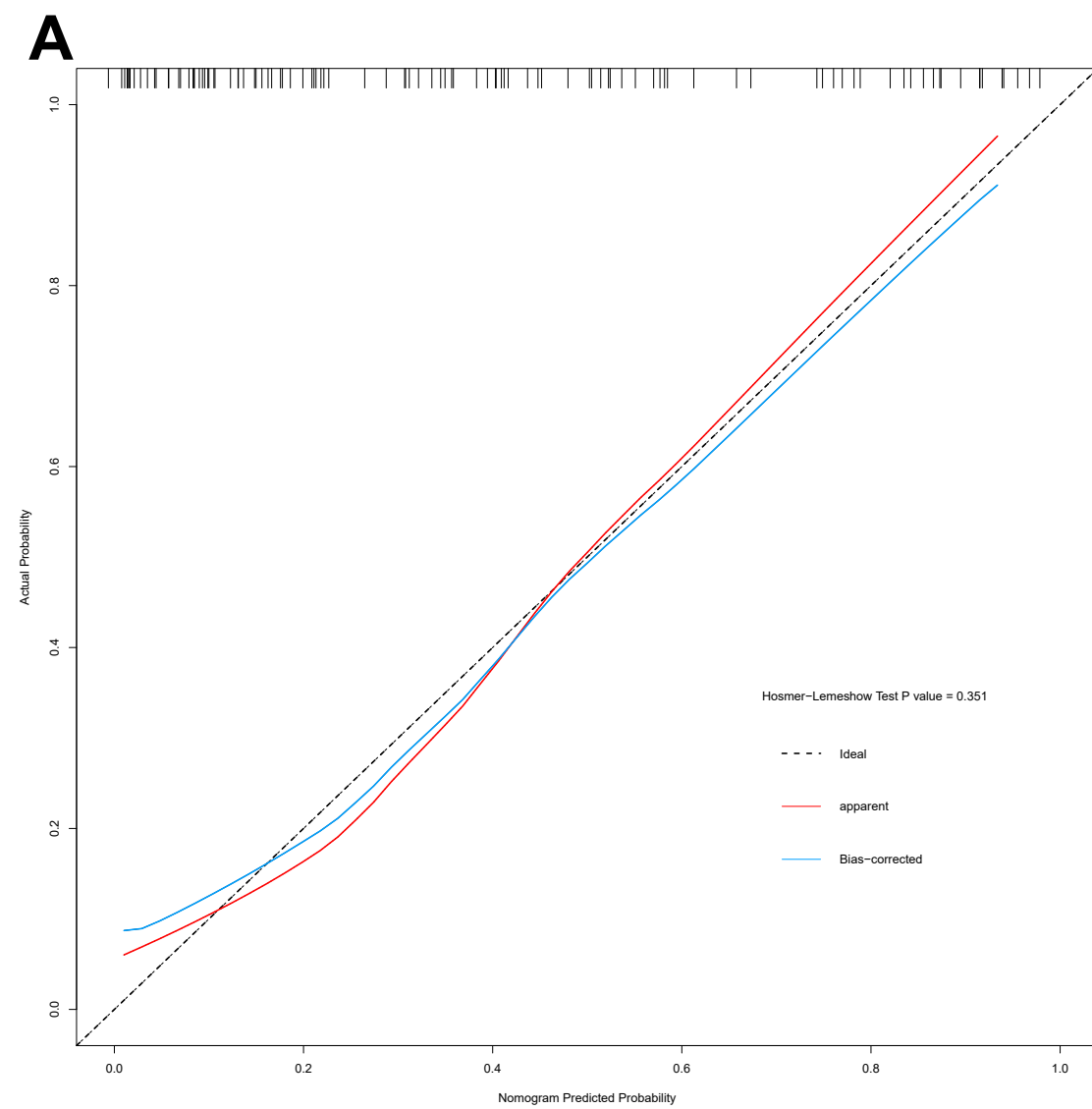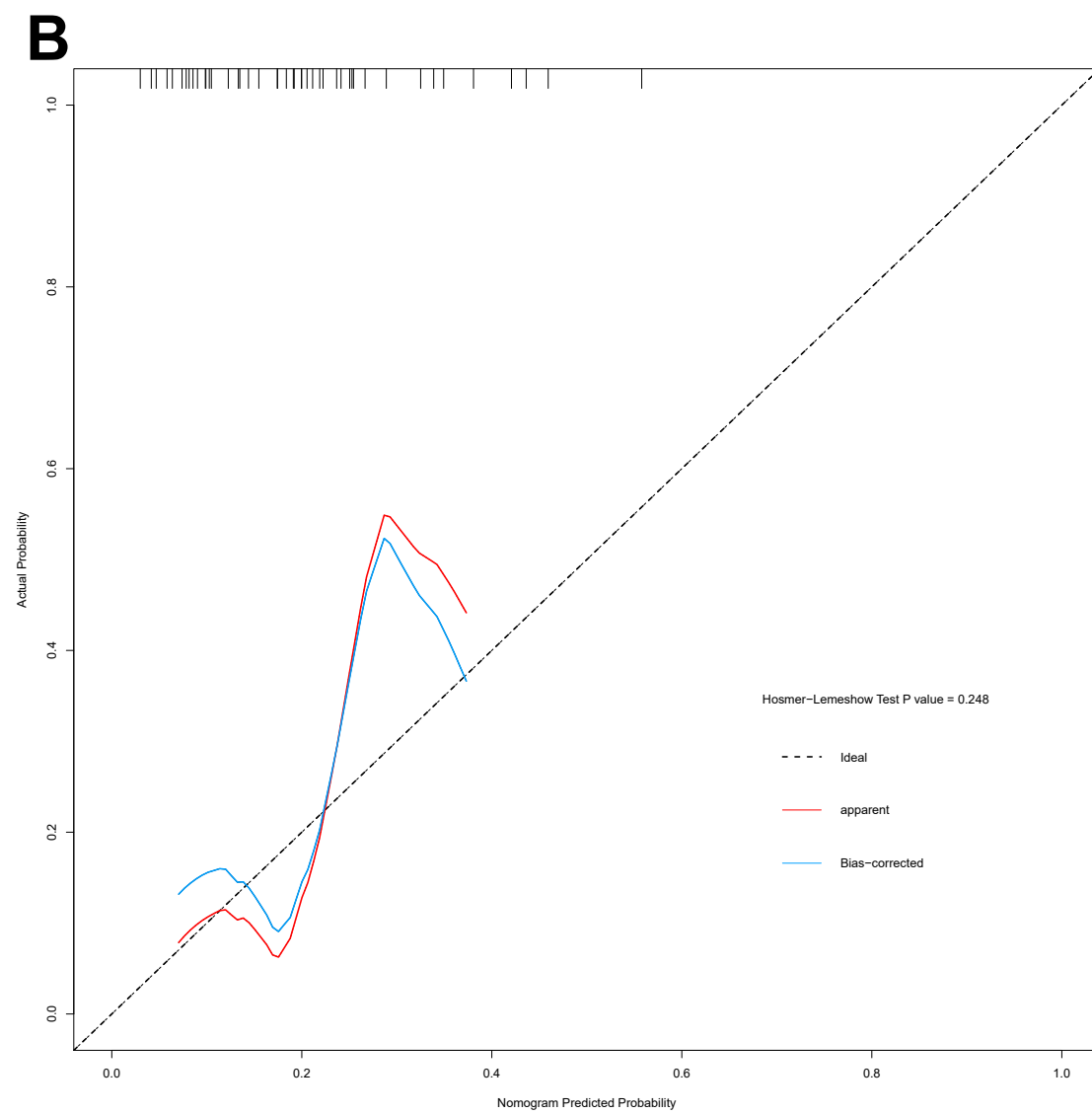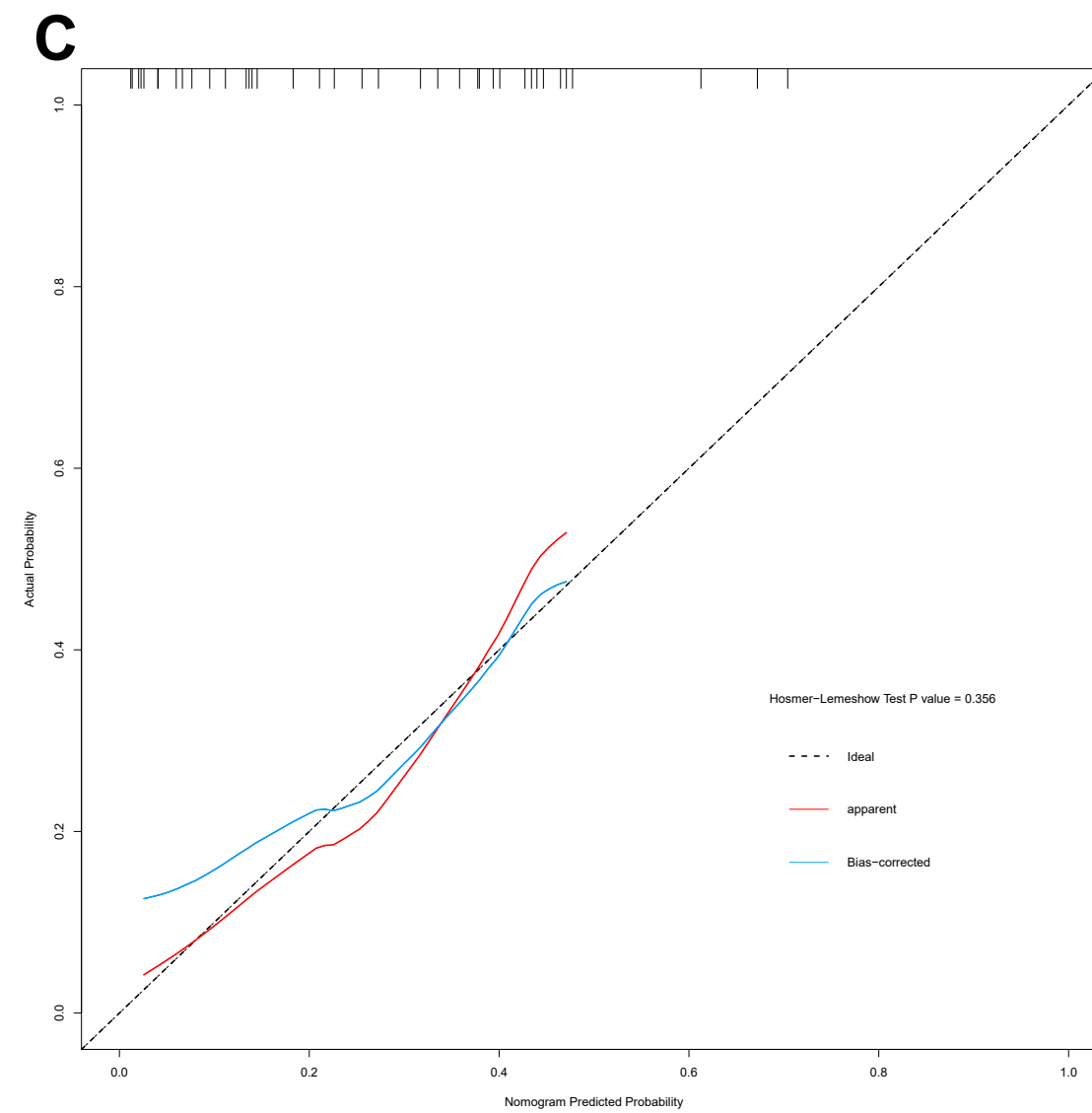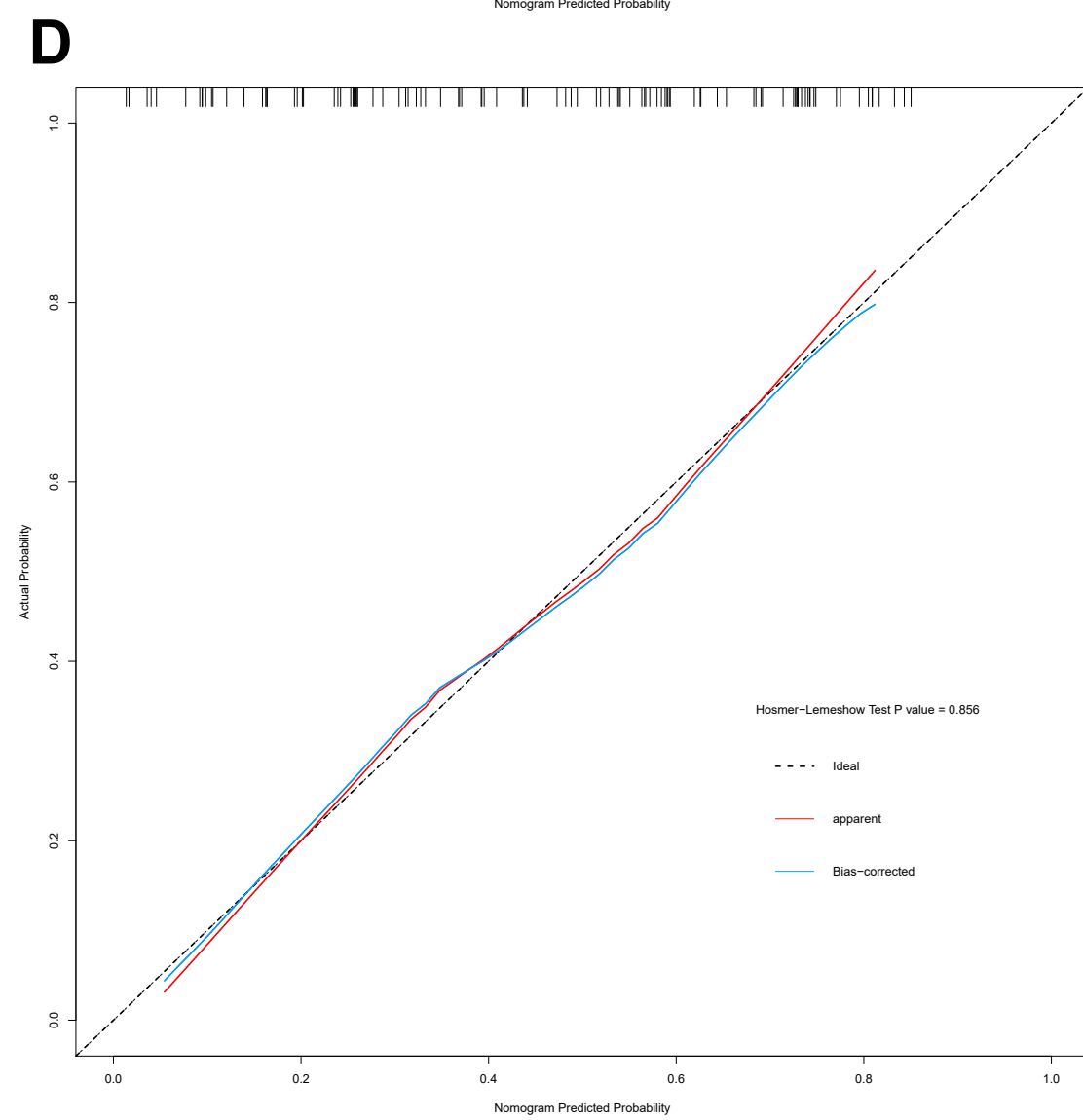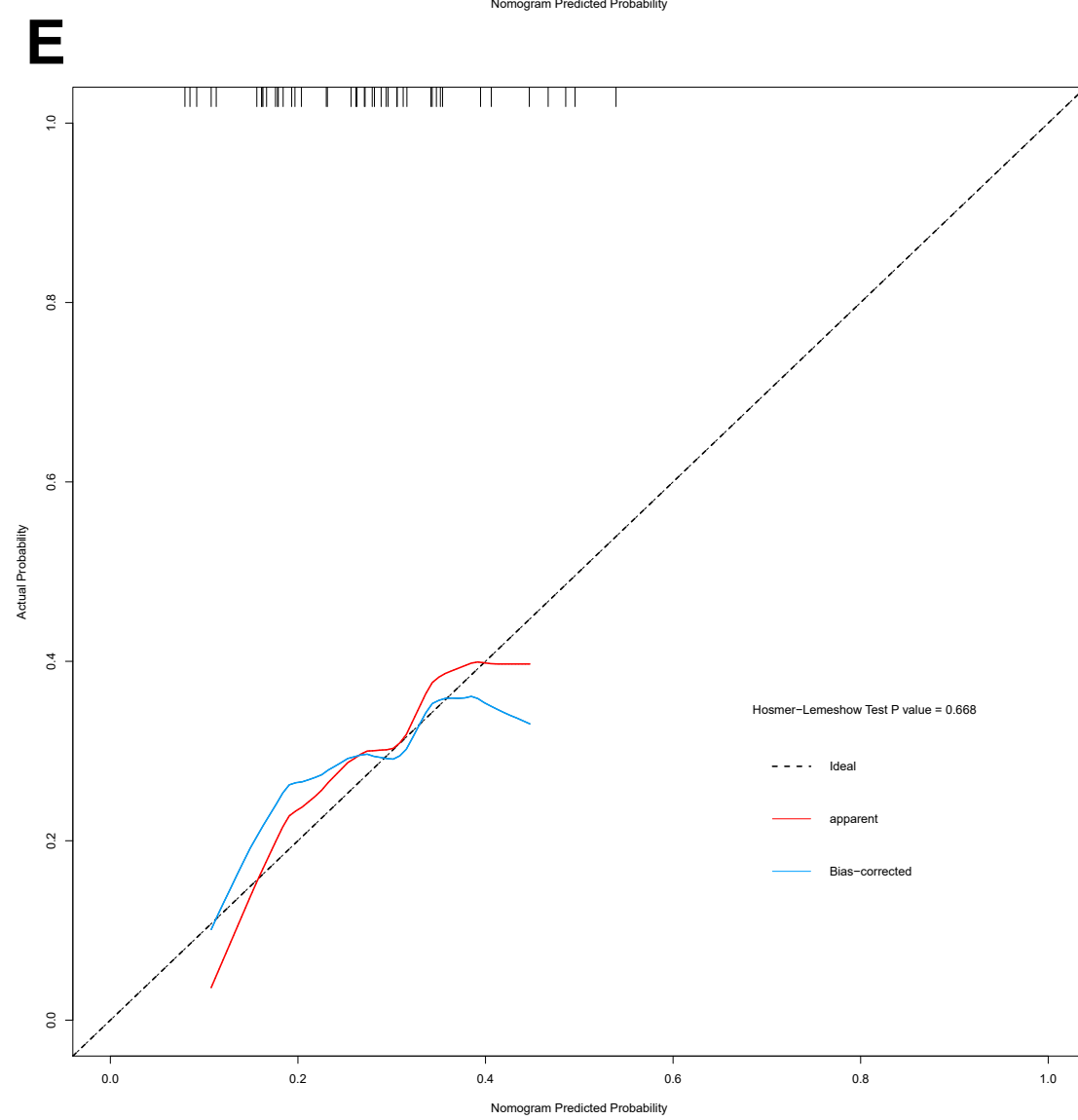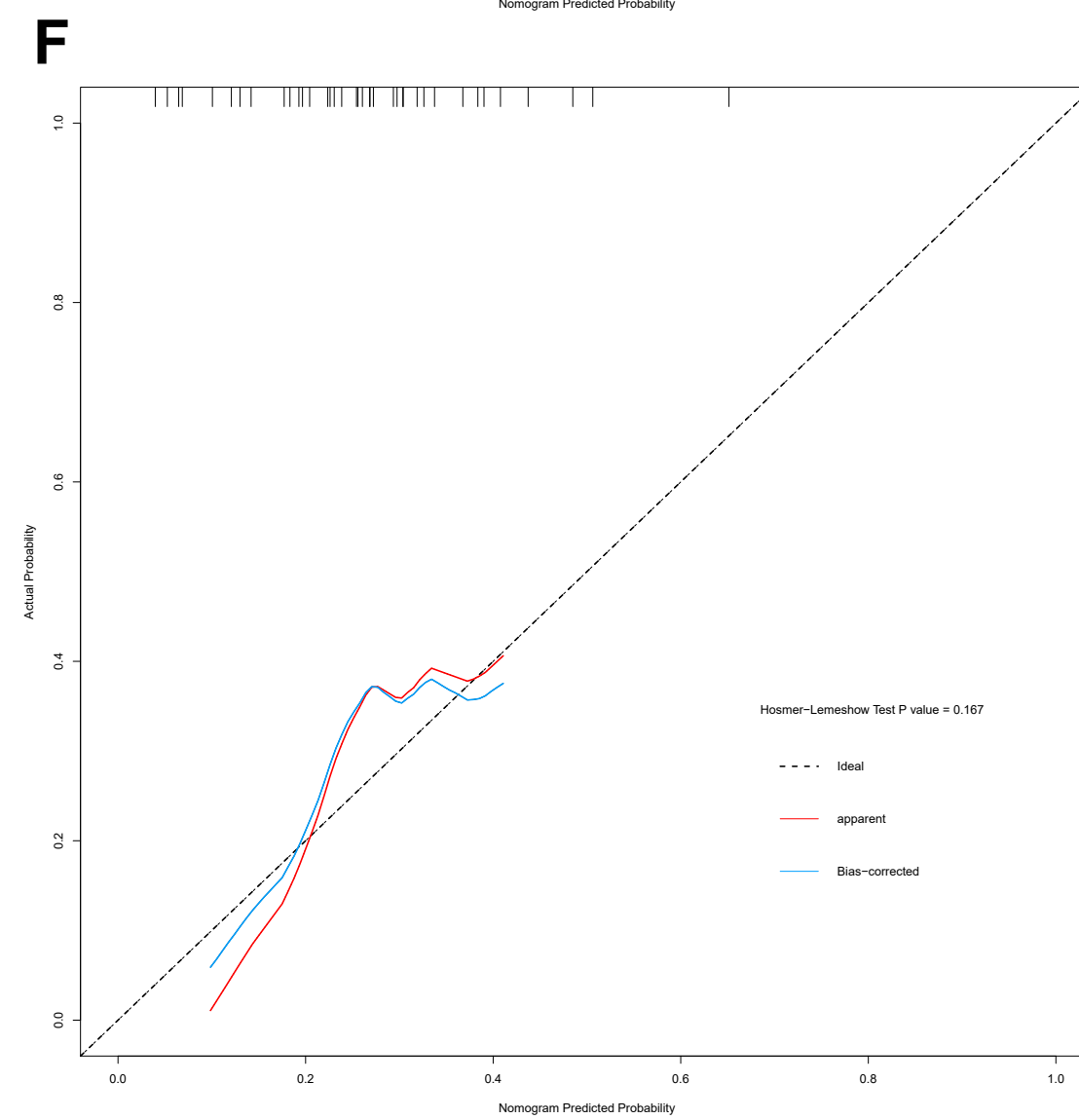

Supplement: Supplementary file 1 — Supplementary Material 1. [file 12957_2024_3474_MOESM1_ESM.pdf]
