# Supplementary material for: Nomograms to predict tumor regression grade (TRG) and ypTNM staging in patients with locally advanced esophageal cancer receiving neoadjuvant therapy
Source: World J Surg Oncol. 2024 Jul 27;22:198. doi: 10.1186/s12957-024-03474-7 (PMC11282666; doi:10.1186/s12957-024-03474-7)
Supplement: Supplementary file 2 — Supplementary Material 2. [file 12957_2024_3474_MOESM2_ESM.docx]

**Supplementary Table 1.** Performance comparison of the ROC curves of independent factors associated with TRG level 0-1 and established prediction model.

| Index | Model | Treatment plan | PFT | PNI | TG | SCC-Ag |
| --- | --- | --- | --- | --- | --- | --- |
| AUC | 0.87 | 0.64 | 0.59 | 0.60 | 0.54 | 0.76 |
| Best Cut-off Value | - | - | - | 52.78 | 66.95 | 1.30 |
| Sensitivity | 87.50 | 55.00 | 67.50 | 80.00 | 55.00 | 82.50 |
| Specificity | 76.92 | 72.31 | 50.77 | 44.62 | 64.62 | 61.54 |
| Negative Predictive Value | 90.91 | 72.31 | 71.74 | 78.38 | 70.00 | 85.11 |
| Positive Predictive Value | 70.00 | 55.00 | 45.76 | 47.06 | 48.89 | 56.90 |
| True Positive Rate | 87.50 | 55.00 | 67.50 | 80.00 | 55.00 | 82.50 |
| False Positive Rate | 23.08 | 27.69 | 49.23 | 55.39 | 35.39 | 38.46 |
| True Negatice Rate | 76.92 | 72.31 | 50.77 | 44.62 | 64.62 | 61.54 |
| False Negative Rate | 12.50 | 45.00 | 32.50 | 20.00 | 45.00 | 17.50 |
| False Discovery Rate | 30.00 | 45.00 | 54.24 | 52.94 | 51.11 | 43.10 |
| Accuracy | 80.95 | 65.71 | 57.14 | 58.10 | 60.95 | 69.52 |
| Precision | 70.00 | 55.00 | 45.76 | 47.06 | 48.89 | 56.90 |
| Youden Index | 164.42 | 127.31 | 118.27 | 124.62 | 119.62 | 144.04 |

ROC, receiver operating characteristic; TRG, tumor regression grade; AUC, area under curve; PFT, pulmonary function tests; PNI, prognostic nutritional index; TG, triglyceride; SCC-Ag, squamous cell carcinoma antigen.
