# Supplementary material for: Nomograms to predict tumor regression grade (TRG) and ypTNM staging in patients with locally advanced esophageal cancer receiving neoadjuvant therapy
Source: World J Surg Oncol. 2024 Jul 27;22:198. doi: 10.1186/s12957-024-03474-7 (PMC11282666; doi:10.1186/s12957-024-03474-7)
Supplement: Supplementary file 3 — Supplementary Figure 1.Calibration curves of the prediction TRG level and ypTNM stage nomograms in the training and validation cohorts. (A) TRG level nomogram calibration curves for training cohorts; (B) TRG level nomogram calibration curves for internal validation cohorts; (C) TRG level nomogram calibration curves for exteral validation cohorts; (D) ypTNM stage nomogram calibration curves for training cohorts; (E) ypTNM stage nomogram calibration curves for internal validation cohorts; (F) ypTNM stage nomogram calibration curves for exteral validation cohorts. nomogram in the training cohort (A), internal validation cohort (B) and external validation cohort (C). [file 12957_2024_3474_MOESM3_ESM.docx]

**Supplementary Table 2.** Performance comparison of the ROC curves of independent factors associated with ypTNM stage I and established prediction model.

| Index | Model | SCC-Ag | PA |
| --- | --- | --- | --- |
| AUC | 0.78 | 0.76 | 0.61 |
| Best Cut-off Value | - | 1.47 | 22.00 |
| Sensitivity | 85.42 | 83.33 | 83.33 |
| Specificity | 57.90 | 59.65 | 43.86 |
| Negative Predictive Value | 82.50 | 80.95 | 75.76 |
| Positive Predictive Value | 63.08 | 63.49 | 55.56 |
| True Positive Rate | 85.42 | 83.33 | 83.33 |
| False Positive Rate | 42.11 | 40.35 | 56.14 |
| True Negatice Rate | 57.90 | 59.65 | 43.86 |
| False Negative Rate | 14.58 | 16.67 | 16.67 |
| False Discovery Rate | 36.92 | 36.51 | 44.44 |
| Accuracy | 70.48 | 70.48 | 61.91 |
| Precision | 63.08 | 63.49 | 55.56 |
| Youden Index | 143.31 | 142.98 | 127.19 |

ROC, receiver operating characteristic; AUC, area under curve; PA, prealbumin; SCC-Ag, squamous cell carcinoma antigen.
